# A Transcriptomic Analysis of Smoking-Induced Gene Expression Alterations in Coronary Artery Disease Patients

**DOI:** 10.3390/ijms241813920

**Published:** 2023-09-10

**Authors:** Mohammed Merzah, Szilárd Póliska, László Balogh, János Sándor, István Szász, Shewaye Natae, Szilvia Fiatal

**Affiliations:** 1 Department of Public Health and Epidemiology, Faculty of Medicine, University of Debrecen, H-4032 Debrecen, Hungary; mohammed.merzah@med.unideb.hu (M.M.); sandor.janos@med.unideb.hu (J.S.); natae.shewaye@med.unideb.hu (S.N.); 2 Department of Biochemistry and Molecular Biology, Faculty of Medicine, University of Debrecen, H-4032 Debrecen, Hungary; 3 Cardiology and Cardiac Surgery Clinic, University of Debrecen, H-4032 Debrecen, Hungary; 4 ELKH-DE Public Health Research Group, Department of Public Health and Epidemiology, Faculty of Medicine, University of Debrecen, H-4032 Debrecen, Hungary

**Keywords:** coronary artery disease, gene expression, smoking, NGS, RNAseq

## Abstract

Smoking is a well established risk factor for coronary artery disease (CAD). Despite this, there have been no previous studies investigating the effects of smoking on blood gene expression in CAD patients. This single-centre cross-sectional study was designed with clearly defined inclusion criteria to address this gap. We conducted a high-throughput approach using next generation sequencing analysis with a single-end sequencing protocol and a read length of 75-cycles. Sixty-one patients with a median age of 67 years (range: 28–88 years) were recruited, and only 44 subjects were included for further analyses. Our investigation revealed 120 differentially expressed genes (DEGs) between smokers and nonsmokers, with a fold change (FC) of ≥1.5 and a *p*-value < 0.05. Among these DEGs, 15 were upregulated and 105 were downregulated. Notably, when applying a more stringent adjusted FC ≥ 2.0, 31 DEGs (5 upregulated, annotated to immune response pathways, and 26 downregulated, involving oxygen and haem binding or activity, with FDR ≤ 0.03) remained statistically significant at an alpha level of <0.05. Our results illuminate the molecular mechanisms underlying CAD, fortifying existing epidemiological evidence. Of particular interest is the unexplored overexpression of *RCAN3*, *TRAV4*, and *JCHAIN* genes, which may hold promising implications for the involvement of these genes in CAD among smokers.

## 1. Background

Coronary artery disease (CAD) is a prevalent chronic condition that affects the arteries responsible for delivering oxygenated blood to the heart muscle [1]. It is primarily caused by atherosclerosis. The latter is characterized by the accumulation of fatty substances in artery walls [2]. As cholesterol, fatty deposits, and calcium build up in the endothelium of arteries, plaque develops and gradually narrows coronary arteries. This severely limits blood flow to the heart and significantly increases the risk of severe ailments such as stroke and myocardial infarction [2]. Clinical manifestations of this process may take years or decades to appear. However, certain risk factors, such as smoking, high cholesterol, high blood pressure, diabetes, obesity, and a sedentary lifestyle, might hasten the onset of atherosclerosis [3]. Previous studies have shown that the chemical compounds in cigarettes can alter the expression of several genes in the endothelial cells of the arteries, further exacerbating the risk of atherosclerosis [4,5,6].

Cigarette smoking is a well established, modifiable risk factor for CAD [7,8]. In Hungary, tobacco consumption, including direct and second-hand smoke, contributes signifi-cantly to mortality. Approximately 21% of all deaths in Hungary are attributed to tobacco use [9]. Despite efforts, smoking rates in the country remain substantial, with around one in four adults reporting daily smoking in 2019, ranking Hungary third highest in Europe [9]. In 2020, tobacco use prevalence reached 28%, surpassing the European average of 25% [9]. This situation is concerning, especially as Hungary grapples with a substantial burden of coronary heart disease, constituting 24.5% of all deaths [9,10].

Emerging research has underscored the pivotal role of inflammation in the initiation, progression, and complications of atherosclerosis. Immune mechanisms are involved in the pathogenesis and clinical manifestation of atherosclerosis [2]. Monocytes, critical players in the immune response, are recruited to the inflamed endothelium, where they transmigrate into the arterial wall. Here, they transform into macrophages and engulf modified low-density lipoproteins, forming characteristic ‘foam cells’ that contribute to forming fatty streaks within the arterial intima [11]. While initially serving as a host defence mechanism, this process ultimately releases pro-inflammatory cytokines and chemokines, further amplifying the inflammatory milieu. Inflammation in atherosclerosis contributes to plaque vulnerability [12]. Plaque rupture exposes the highly thrombogenic material to circulating platelets, triggering the formation of a thrombus that can occlude the artery, leading to acute cardiovascular events such as stroke or myocardial infarction [12].

In the development of atherosclerosis, numerous blood cells are involved, and many of these cells can be found in the whole peripheral blood, including B cells, natural killer cells, T cells, white blood cells, platelets, and circulation stem cells [11,12]. Given the role of these cells in atherosclerosis, examining gene expression in whole blood using next-generation sequencing (NGS) techniques can provide valuable insights into the mechanisms underlying this condition [12].

Despite the well-established link between smoking and CAD [7,13,14,15], to our knowledge, no previous studies have examined the effects of smoking on the blood transcriptome of CAD patients. Therefore, the current study aims to address this gap in the literature by conducting a transcriptome analysis to describe the global gene expression in the whole blood of smokers and nonsmokers among CAD patients in Hungary. By identifying and comparing differentially expressed genes based on smoking status, the study seeks to contribute to our understanding of the molecular mechanisms underlying the effects of smoking on CAD in this population. Additionally, the findings of this study could potentially strengthen the existing epidemiological evidence that highlights the influence of smoking on the prevalence and occurrence of CAD.

## 2. Results

The study included sixty-one participants, with 31 (50.8%) smokers and 30 (49.2%) nonsmokers. The median age of smokers was significantly lower than that of nonsmokers (60 vs. 75 years, *p* < 0.001). The proportion of male participants was higher among smokers than nonsmokers (37.7% vs. 19.7%), while the proportion of female nonsmokers was higher than smokers (29.5% vs. 13.1%). The difference in gender proportions was statistically significant (*p* = 0.007).

The proportion of smokers with a family history of CAD was significantly higher than that of nonsmokers (45.2% vs. 20%, *p* = 0.04). The characteristics of the study participants are presented in Table 1.

Quantitative analysis of gene expression profiles using the moderated t test was performed to identify and compare the differentially expressed genes (DEGs) between smokers and nonsmokers, revealing distinct molecular signatures associated with each group. A total of 502 genes were significant in their expression with a *p*-value <0.05, but only 13 genes remained after adjusting the FC to ≥1.5 (Supplementary Table S1).

Further analyses were performed after excluding 17 samples, as they were considered outliers based on principal component analysis and clustering on the heatmap. With the remaining 44 samples (the characteristics described in Supplementary Table S2), our analysis revealed 120 DEGs between the smoker and nonsmoker groups, with a *p*-value < 0.05 and an FC of ≥1.5 (Supplementary Table S3 and Supplementary Figure S1). Of these, 15 genes were upregulated, and 105 genes were downregulated. Table 2 lists the top 10 upregulated and downregulated genes, including their fold change values and associated *p*-values. Notably, the GPR15 gene was upregulated by 7-fold with a *p*-value <0.001, while the KRT1 gene was downregulated by 3-fold with a *p*-value of 0.006.

**Figure 1:**
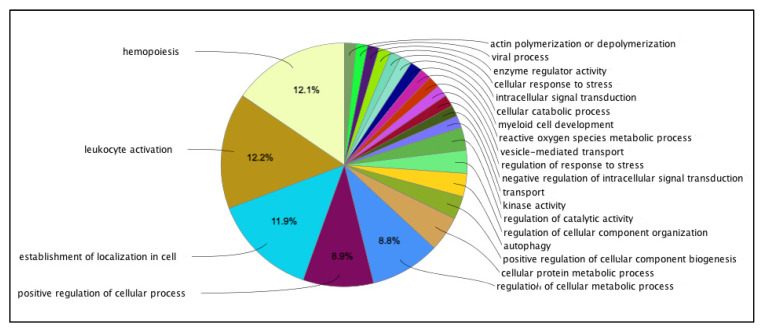
Gene ontology (GO) enrichment analysis of the most significant pathways. All the depicted pathways were significant at an FDR value of <0.001. Each slice represents the relative proportion of the identified significant genes associated with a given GO term.

Gene ontology (GO) analysis revealed that the upregulated genes were primarily involved in the immune response and inflammation pathways. In contrast, the downregulated genes were primarily involved in reactive oxygen species metabolic processes (Figure 1). The analysis of GO functions of the upregulated genes identified 18 significant biological processes and 3 cellular components with a corrected *p*-value of <0.05 (Supplementary Table S4). For the downregulated genes, 14 biological processes, 5 molecular functions, and 5 cellular components were identified (Supplementary Table S5).

A pathway analysis was performed using the KEGG database, which revealed significant enrichment of immune response and inflammation pathways in the upregulated gene set, such as the haematopoietic cell lineage pathway (FDR = 0.01) and the arrhythmogenic right ventricular cardiomyopathy pathway (FDR = 0.01). In contrast, the downregulated gene set showed significant enrichment of metabolic pathways and porphyrin and chlorophyll metabolism pathways (FDR = 0.03) (Supplementary Table S6).

**Figure 2:**
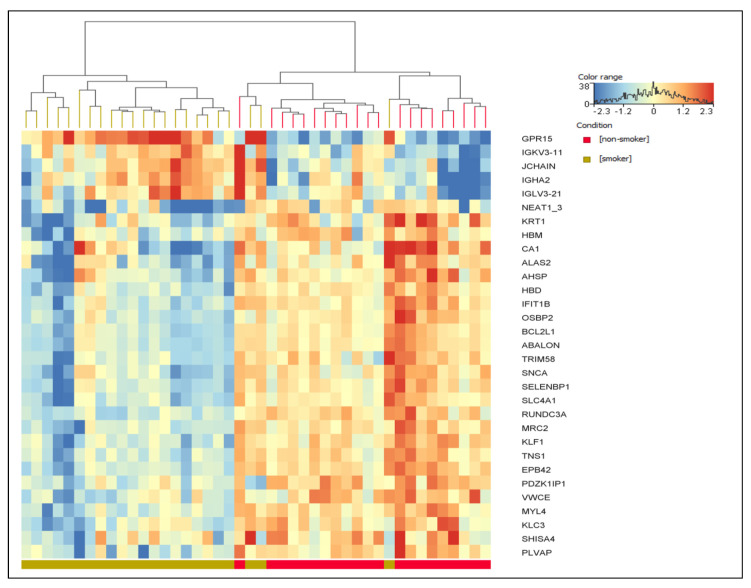
Heatmap of the differentially expressed genes with a fold change of ≥2.

When the FC was increased to ≥2.0 with an adjusted *p*-value of <0.05, 31 DEGs remained significant, including 5 upregulated and 26 downregulated genes (Table 3). We also generated a heatmap of the 31 DEGs (Figure 2), showing the separation between the smoker and nonsmoker groups based on their gene expression profiles. The heatmap also highlights the upregulation of genes related to the immune response and inflammation pathways and the downregulation of genes related to oxygen binding and activity pathways.

A total of 27 DEGs were identified when comparing subjects according to their ND status: high, moderate, and low to moderate ND were compared to low ND using an FC ≥1.5 threshold and a *p*-value of <0.05. Notably, nine genes (*ADPGK-AS1, ASB16, IGKV1-12, RN7SL2, SCGB3A1, SPTLC1P2, UBE2SP2, USP9Y,* and *ZNF696*) were consistently overexpressed across all comparison groups (Supplementary Figure S2 and Supplementary Table S7). GO analyses revealed that upregulated genes were associated with immune response activities, while the downregulated genes were annotated to receptor regulator, inhibitor, and antagonist activities (Supplementary Figure S3).

## 3. Discussion

This analysis demonstrated that cigarette smoking significantly impacted the expression of genes related to the immune response and oxygen binding. The study revealed that 120 genes were differentially expressed with an FC of ≥1.5. Although this is a cross-sectional study, the findings might reinforce epidemiological observations regarding the impact of cigarette consumption on CAD. By providing additional evidence, these findings support the hypothesis that cigarette consumption may have a detrimental impact on CAD. However, it is essential to note that while changes in gene expression have been observed in peripheral blood (contacting mainly leukocytes and lymphocytes), these changes alone do not definitively establish a causal relationship with CAD. Further research is required to elucidate the potential mechanistic links between cigarette consumption, gene expression changes, and the development or progression of CAD.

Smoking is a recognized independent risk factor influencing CAD severity and pattern [16]. A study on the relationship between smoking and the risk of coronary heart disease (CHD) in various age groups demonstrated that most CHD cases are attributable to smoking across all age ranges. However, the youngest participants had the highest attribution, with a hazard ratio of 8.5 (95% CI 5.0–14.0) among women aged 40 to 49 years [17]. Our study revealed that smokers exhibited a lower mean age compared to nonsmokers. This finding aligns with recent studies that revealed smoking as a prominent risk factor for the premature onset of cardiovascular disease [18,19]. In addition, our investigation revealed a high prevalence of CAD in males than females, a trend supported by other studies [20,21]. The age and gender difference observed between nonsmokers and smokers raises a valid concern regarding the potential influence of age and gender on the outcomes measured in our study. Future research using better age- and gender-matched groups may clarify the independent effects of smoking on gene expression among CAD patients.

### 3.1. Link of Upregulated Genes to CAD and/or Smoking

CAD is primarily caused by atherosclerosis, involving numerous inflammatory processes. Our analyses revealed 15 upregulated genes, all of which are reportedly involved in regulating the immune response. Six of them were related to immunoglobulin (Ig) receptors or secretions, including *IGHA2, IGKV3-11, IGLC1, IGLL5, IGLV3-21,* and *JCHAIN*. In general, Igs, known as antibodies, are a group of heterodimeric glycoproteins produced by B-lymphocytes [22]. The membrane-bound Igs act as receptors. Upon binding to a particular antigen, they trigger B-lymphocyte replication and differentiation, resulting in Ig-secreting plasma cells. These secreted Igs are crucial in facilitating the effector phase of humoral immunity [22]. There is evidence that the processes of plaque development are modulated by humoral immunity [11]. In agreement with our findings, a study found that smoking differentially affected IgM and IgA concentrations, leading to an elevation in their serum levels [23]. Another study additionally revealed that measuring the plasma level of *IGHA2*, encoded by IgA, might be a valuable biomarker in detecting subclinical atherosclerosis [24]. Similarly, the expression levels of *CD28*, *TRAT1*, *TRAV13-1*, and *LEF1* were related to smoking and smoking-related atherosclerosis [25,26,27,28]. Those genes play a central role in the activation of adaptive immunity represented by T-cell activation, which is the major driver of lesion formation [11].

The significance of *GPR15*, a chemokine receptor known as G protein-coupled receptor 15, has been emphasized as a vital regulator of T-cell trafficking in systemic inflammation. Current studies have demonstrated that increased expression of *GPR15* is associated with cardiovascular disorders, mediating the negative consequences of smoking [29,30]. The significantly increased expression (7.5 times in smokers vs. nonsmokers) of *GPR15* in our analysis might indicate a host immunological response to counteract the effects of smoking. Correspondingly, smoking has been reported to be significantly associated with the upregulation of *CCR7* [31], a gene that encodes a protein called C–C chemokine receptor type 7, and the promotion of T-cell trafficking in CAD patients [32].

The *ITGA6* gene, a member of the integrin family, plays a crucial role in multiple biological processes, including cell proliferation, adhesion, and invasion [33]. The higher expression of *ITGA6* was found to significantly influence macrophages in driving atheroma formation [34]. According to Scott and Palmer, it has been proposed that smoking influences the profile of circulating adhesion molecules. Smoking can lead to elevated concentrations of soluble adhesion molecules, which may serve as an indicator of ongoing inflammatory processes that play a crucial role in atheroma formation [35].

*RCAN3* is a coding protein expected to play a role in calcium-mediated signalling pathways. No specific studies have examined the relationship between *RCAN3* and CAD or smoking, except for a single study that demonstrated a negative association between DNA methylation and the risk of myocardial infarction [36]. However, since this gene is involved in calcium-mediated processes, a recent study proposed a novel mechanism of increased atherosclerosis calcification through the induction of intracellular calcium mediated by nicotine [37]. This mechanism could potentially provide a plausible rationale for the upregulated *RCAN3* gene among smokers with CAD. Furthermore, *TRAV4*, which encodes T-cell receptor alpha variable 4, and *JCHAIN*, which encodes Ig J Chain, have been reported to be related to adenocarcinoma [38,39]. However, no previous studies have examined their connection to CAD or smoking. Given their involvement in adaptive immunity, they might contribute to their overexpression in this study.

### 3.2. Pathways of the Downregulated Genes

Instead of delving into an overwhelming list of over a hundred downregulated genes, we focused more on the associated GO terms. Our study revealed significant molecular functions, including oxygen carrier activity, oxygen binding, haemoglobin binding, organic acid binding, and haem binding. Six genes were found to share these molecular functions: *HBQ1, HBA2, HBA1, HBB, HBM,* and *HBD*. Haemoglobin subunit beta (*HBB*) and delta (*HBD*) genes play a critical role in the proper functioning of haemoglobin, which is responsible for oxygen transport in the blood [40]. Smoking can restrict the oxygen transport capacity of haemoglobin [41], which may be attributed to the downregulation of these genes. On the other hand, haemoglobin subunit α2 (*HBA2*) is a paralogue of α1 (*HBA1*), with the latter being an important paralogue of theta1 (*HBQ1*). These genes were reported to be related to erythrocyte function, particularly carbon dioxide uptake and oxygen delivery. As a result of smoking, this phenomenon might reduce oxygen availability in CAD patients [42].

Furthermore, alpha haemoglobin stabilizing protein (*AHSP*), a small protein that regulates the stability and folding of the alpha-globin subunit, was downregulated, possibly due to impaired oxygen transport in CAD patients due to smoking. Similarly, the *SLC4A1 gene*, a member of soluble carrier family 4, has been observed to be downregulated and identified as one of the genes affecting haemoglobin-binding activity. A recent study has shown that oxidative stress (OS) can influence the expression of *SLC4A1*, and these effects could have negative implications for diseases associated with OS [43], such as CAD. As the evidence linking smoking and OS continues accumulating, this connection could potentially explain the downregulation of the *SLC4A1* gene in CAD patients who smoke.

Notably, carbon dioxide in cigarettes, along with other harmful chemicals, exacerbates the adverse health effects of smoking [4,5]. Continued exposure to these chemicals can lead to OS [5], which impairs regular gene expression and leads to the downregulation of genes involved in various physiological processes, such as oxygen and haem binding or carrier activity [44].

The protein produced by the *ALAS2* gene, known as 5’-aminolevulinate synthase 2, plays a role in initiating the haem biosynthetic pathway [45]. Emerging evidence indicates that this protein potentially acts as a mediator by connecting smoking with IL6 and CRP, both of which are inflammatory markers, and exhibiting an inverse association [46]. The low expression of this gene might be attributed to smoking-related inflammation. Similarly, *PTGDS* is a gene that encodes a protein involved in prostaglandin D synthesis. It functions in the relaxation and contraction of smooth muscle cells and platelet aggregation. Consistent with our findings, studies have demonstrated that the *PTGD* gene is downregulated in smokers and associated with smoking and plaque development [47,48]. A mouse model also highlighted the involvement of *PTGDS* in promoting thrombosis [47]. Furthermore, impaired *PTGDS* expression could lead to the accumulation of lymphocytes and macrophages, accelerating the development of atherosclerosis [48]. Ultimately, according to our analysis, these genes (*HBQ1, HBA2, HBA1, ALAS2, HBB, HBM, PTGDS,* and *HBD*) were involved in organic acid binding pathways. Further in-depth studies are required to investigate their potential impact on the development and progression of CAD in individuals who smoke. Understanding the intricate connection between smoking, CAD, and organic acid binding is crucial for comprehending the underlying mechanisms and exploring potential therapeutic targets.

### 3.3. Limitations

Patients’ referral and admission processes in this study were subject to a significant limitation: the time required for completion of those processes exceeded the half-life of plasma cotinine, which is 16–17 h [49]. Consequently, cotinine became undetectable for most smokers within that time frame. Therefore, the study relied on self-reported smoking status and the Fagerström standard for nicotine dependence [50] to address this limitation.

The age variance might introduce age- or gender-related bias, potentially affecting the interpretation of our results. Despite efforts to control for confounding variables, the inherent age and gender difference could influence outcomes. Future studies involving more precisely age-and gender-matched cohorts could clarify the distinct impact of smoking on gene expression in individuals with coronary artery disease. Additionally, patients with coronary stenosis of ≥50% of the luminal diameter were included based on angiography, subject to somewhat subjective interpretation by the intervention cardiologist. This approach has been commonly utilized in previous studies as the established method for diagnosing CAD; however, invasive intravascular ultrasound methods could provide a more quantitative evaluation for CAD diagnosis.

This analysis seeks to identify the gene expression implicated in atherogenesis using whole peripheral blood instead of atherosclerotic plaques. Atherosclerosis is a multifaceted disease that involves the interplay between circulating blood and the endothelium. Hence, relying solely on blood circulation may only capture some aspects of the intricate pathophysiology of atherogenesis. Thus, utilizing intracoronary or coronary sinus sampling may be the more appropriate approach.

## 4. Materials and Methods

### 4.1. Study Design and Population

A single-centre cross-sectional study was conducted between November 2021 and October 2022 at the Cardiology and Cardiac Surgery Clinic, University of Debrecen Clinical Centre. 

To determine the appropriate sample size for this study, we employed the MD-Anderson Bioinformatics website, which facilitates the computation of sample sizes for genomic experiments [51]. Ultimately, a sample size of 60 patients was selected to ensure a power of 0.8 for detecting 1.5-fold differences with a two-tailed alpha level of 0.05. Sixty-one patients were recruited for this study based on specific inclusion and exclusion criteria. Adult patients who underwent coronary angiography for the first time and had documented coronary lesions (>50% of the original diameter of the affected artery/ies) were included. Patients with hypertension, obesity, diabetes mellitus, lung inflammation, stroke, prior coronary revascularization, or a luminal diameter <50% of the original diameter of the affected artery were excluded.

### 4.2. Blood Sample Collection

Peripheral venous whole blood samples were collected from all study participants using EDTA tubes for haematological tests and cotinine assays and PAXgene^®®^ tubes (PreAnalytiX, Hombrechtikon, Switzerland; reference number: 762165) for RNA analyses. After sampling, PAXgene tubes were incubated at room temperature for two hours and then stored at −80 °C until use. Plasma was also collected from EDTA tubes within 30 min of blood withdrawal and stored in aliquots at −80 °C for subsequent analysis.

### 4.3. RNA Isolation, Library Preparation, and Sequencing

Following the manufacturer’s instructions, total RNA was extracted from blood samples using the PAXgene Blood RNA Kit (PreAnalytiX, Hombrechtikon, Switzerland). RNA quality was assessed using the Eukaryotic Total RNA Nano Assay on the Agilent 2100 bioanalyzer according to the manufacturer’s protocol (Agilent Technologies, Santa Clara, CA, USA) [52]. Samples with an RNA integrity number ≥7 were accepted for the library preparation process.

RNA sequencing libraries were prepared using the NEBNext Ultra II RNA Library Prep Kit (New England Biolabs, Ipswich, MA, USA) following the manufacturer instructions. Briefly, poly-A RNAs were captured by oligo-dT-conjugated magnetic beads, and then the mRNAs were eluted and fragmented at 94 degrees Celsius. First-strand cDNA was generated by random priming reverse transcription, and after the second-strand synthesis step, double-stranded cDNA was generated. The cDNA fragments were then subjected to end repair, A-tailing, and adapter ligation. PCR amplification was performed to enrich adapter-ligated fragments, and sequencing libraries were generated. RNA sequencing was performed on an Illumina NextSeq 500 instrument using a single-end sequencing protocol with a read length of 75 cycles.

### 4.4. Cotinine Assay

Cotinine concentrations were assessed using an ELISA Kit for Cotinine assay, following the manufacturer’s guidelines (ELISA Kit for Cotinine, Cloud-Clone Corp., Wuhan, China; Reference number: CET058Ge). Absorbance was measured at 450 nm using an Epoch™ Microplate Spectrophotometer (BioTek Instruments, Inc., Winooski, VT, USA). A <1 ng/mL cotinine concentration was specified for nonsmokers and ≥10 ng/mL for active smokers [53].

### 4.5. Data Collected

Demographic data such as age, gender, laboratory data (total cholesterol, triglyceride, HDL-C, LDL-C), family history of CAD, smoking status and nicotine dependence, BMI, blood pressure, and data on anticoagulation therapy were also collected from each participant. A cardiologist utilized quantitative coronary angiography to estimate the percentage of coronary stenosis for each study participant. Luminal diameters less than 50% of the original diameter were excluded from the study, while those with 50–70% and greater than 70% were classified as moderate and severe stenosis, respectively [54]. The Fagerström Test for Nicotine Dependence was used to measure nicotine dependence (ND) among smokers. The questionnaire has six items with a possible score range of 1–10: Subjects scored ≥ 8 have high ND, 5–7 have moderate ND, 3–4 have low to moderate ND, and ≤2 have low ND [50].

### 4.6. Statistical Analysis

Descriptive statistics for continuous and numerical variables were performed using measures of central tendency (i.e., mean, standard deviation, and median) when appropriate, while categorical variables were expressed as absolute values or relative frequencies. All statistical analyses were conducted using the R software package.

Quality control of all throughput sequencing data was performed using FastQC (Babraham Bioinformatics, Cambridge, UK) [55]. After trimming the ends of the sequences, the high-quality reads were aligned to the human reference genome (GRCh38) using the HISTAT2 algorithm; BAM files were generated. Downstream analysis was performed using Strand NGS software (www.strand-ngs.com (accessed on 20 August 2023)). The DESeq algorithm of Strand NGS was used for normalization. A moderated t test was used to determine the differentially expressed genes between conditions. Further analyses were conducted among smokers only, where the Kruskal-Wills test was employed to identify the DEGs based on ND.

For identifying overrepresented Gene Ontology (GO) terms, CytoScape (v3.4) and ClueGO (v2.3.5) applications were used. Our approach involved using a list of differentially expressed genes and the GO Biological Process Database. We conducted a two-sided hypergeometric test to assess statistical significance, which was further adjusted using the Benjamini-Hochberg false discovery rate (FDR) correction.

## 5. Conclusions

Our analysis sheds light on the potential impact of cigarette smoking on gene expression in whole blood and their association with smoking-related CAD. GO analyses revealed that the upregulated genes were associated with immune response pathways. In contrast, downregulated genes showed significant annotations with pathways involving oxygen and haem binding or activity. Remarkably, our study revealed a novel finding, as no previous literature has explored the association of *RCAN3, TRAV4*, and *JCHAIN* with smoking or CAD. These genes exhibited overexpression in our study, suggesting potential roles in the pathogenesis of CAD among smokers.

Our results expand the understanding of the genetic factors associated with CAD and highlight the need for further investigation into the functional implications and therapeutic potential of these novel genes. Additionally, the findings of this study may identify blood biomarkers that could serve as targets for the treatment or prevention of health issues related to smoking.

The demand for blood biomarkers to diagnose CAD is growing, as this might provide a precise diagnosis instead of relying solely on angiography and imaging. Future research on the identified genes would be beneficial in identifying blood markers and exploring potential targeted therapies through personalized medication. This approach might ensure that the prescribed medication aligns with the patient’s gene expression profiles.

## Figures and Tables

**Table 1:** Characteristics of study participants (N = 61).

Variables	Total	Smoking Status		*p*-Value
		Smokers	Nonsmokers	
Gender, *n* (%)				
**Male**	**34 (57.4)**	**23 (74.2)**	**12 (40.0)**	**0.007**
**Female**	**26 (42.6)**	**8 (25.8)**	**18 (60.0)**	
**Age, median (range) years**	**67 (28–88)**	**60 (28–75)**	**75 (54–88)**	**<0.001**
Cholesterol (mmol/L), mean (SD)	5.0 (1.4)	5.2 (1.5)	4.8 (1.4)	NS
Triglyceride (mmol/L), mean (SD)	1.6 (2.0)	1.9 (2.7)	1.4 (0.7)	NS
HDL-C (mmol/L), mean (SD)	1.3 (0.6)	1.4 (0.7)	1.3 (0.3)	NS
LDL-C (mmol/L), mean (SD)	3.0 (1.2)	3.1 (1.3)	2.9 (1.2)	NS
**Family history of CAD, *n* (%)**	**20 (32.8)**	**14 (45.2)**	**6 (20.0)**	**0.04**
Anticoagulation medication, *n* (%)	61 (100)	31 (100)	30 (100)	NS
BMI, mean (SD)	26.0 (3.1)	25.3 (3.4)	26.8 (2.5)	NS
Blood pressure (mmHg), mean (SD)				
Systolic	125.9 (22.4)	120.8 (21.9)	131.3 (22.0)	NS
Diastolic	73.3 (12.0)	71.9 (13.5)	74.8 (10.4)	NS
Plasma cotinine ng/mL, mean (SD)	0.3 (0.1)	0.4 (0.2)	0.2 (0.1)	NS
Angiography findings, *n* (%)				
>2 arteries stenosis	45 (73.8)	20 (64.5)	25 (83.3)	NS
Number of affected arteries, median (range)	2 (1–3)	2 (1–3)	2 (1–3)	NS
LAD	50 (82.0)	24 (77.4)	26 (86.6)	NS
LM	10 (16.4)	6 (19.4)	4 (13.3)	NS
**CX**	**34 (55.7)**	**13 (42.0)**	**21 (70.0)**	**0.03**
RCA	35 (57.4)	17 (54.8)	18 (60.0)	NS

N = Number of subjects; NS = Nonsignificant; SD = Standard deviation; LAD = Left anterior descending artery; LM = Left main coronary artery; CX = Circumflex artery; RCA = Right coronary artery. Bold text indicates significant differences between study groups.

**Table 2:** The top 10 upregulated and downregulated genes (FC ≥ 1.5, N = 44).

No.	Gene Symbol	FC	*p*-Value *	Description
**Upregulated genes**				
1	*GPR15*	7.5	1.4 × 10^−9^	G protein-coupled receptor 15 [Source:HGNC Symbol;Acc:HGNC:4469]
2	*IGHA2*	2.6	0.034	immunoglobulin heavy constant alpha 2 (A2m marker) [Source:HGNC Symbol;Acc:HGNC:5479]
3	*IGLV3-21*	2.4	0.030	immunoglobulin lambda variable 3–21 [Source:HGNC Symbol;Acc:HGNC:5905]
4	*IGKV3-11*	2.2	0.006	immunoglobulin kappa variable 3–11 [Source:HGNC Symbol;Acc:HGNC:5815]
5	*JCHAIN*	2.1	0.037	joining chain of multimeric IgA and IgM [Source:HGNC Symbol;Acc:HGNC:5713]
6	*IGLC1*	2.0	0.044	immunoglobulin lambda constant 1 [Source:HGNC Symbol;Acc:HGNC:5855]
7	*IGLL5*	1.9	0.046	immunoglobulin lambda like polypeptide 5 [Source:HGNC Symbol;Acc:HGNC:38476]
8	*LEF1*	1.7	0.001	lymphoid enhancer binding Factor 1 [Source:HGNC Symbol;Acc:HGNC:6551]
9	*CCR7*	1.6	0.030	C-C motif chemokine receptor 7 [Source:HGNC Symbol;Acc:HGNC:1608]
10	*CD28*	1.6	0.002	CD28 molecule [Source:HGNC Symbol;Acc:HGNC:1653]
**Downregulated genes**				
1	*KRT1*	−2.9	0.006	keratin 1 [Source:HGNC Symbol;Acc:HGNC:6412]
2	*NEAT1_3*	−2.7	0.005	Nuclear enriched abundant transcript 1 conserved region 3 [Source:RFAM;Acc:RF01957]
3	*PDZK1IP1*	−2.6	0.044	PDZK1 interacting protein 1 [Source:HGNC Symbol;Acc:HGNC:16887]
4	*PLVAP*	−2.6	8.1 × 10^−6^	plasmalemma vesicle associated protein [Source:HGNC Symbol;Acc:HGNC:13635]
5	*CA1*	−2.4	7.2 × 10^−4^	carbonic anhydrase 1 [Source:HGNC Symbol;Acc:HGNC:1368]
6	*VWCE*	−2.4	0.036	von Willebrand factor C and EGF domains [Source:HGNC Symbol;Acc:HGNC:26487]
7	*KLF1*	−2.4	8.1 × 10^−4^	Kruppel-like factor 1 [Source:HGNC Symbol;Acc:HGNC:6345]
8	*HBM*	−2.3	2.3 × 10^−4^	haemoglobin subunit mu [Source:HGNC Symbol;Acc:HGNC:4826]
9	*AHSP*	−2.3	0.016	alpha haemoglobin stabilizing protein [Source:HGNC Symbol;Acc:HGNC:18075]
10	*EPB42*	−2.2	0.006	erythrocyte membrane protein band 4.2 [Source:HGNC Symbol;Acc:HGNC:3381]

FC = Fold change; negative sign means overexpressed in nonsmokers. * corrected *p*-value.

**Table 3:** Differentially expressed genes with a fold change of ≥2 (N = 44).

No.	Gene Symbol	FC	*p*-Value *	Description
**Upregulated genes**				
1	*GPR15*	7.5	1.4 × 10^−9^	G protein-coupled receptor 15 [Source:HGNC Symbol;Acc:HGNC:4469]
2	*IGHA2*	2.6	2.8 × 10^−2^	immunoglobulin heavy constant alpha 2 (A2m marker) [Source:HGNC Symbol;Acc:HGNC:5479]
3	*IGKV3-11*	2.2	6.2 × 10^−3^	immunoglobulin kappa variable 3–11 [Source:HGNC Symbol;Acc:HGNC:5815]
4	*IGLV3-21*	2.4	3.0 × 10^−2^	immunoglobulin lambda variable 3–21 [Source:HGNC Symbol;Acc:HGNC:5905]
5	*JCHAIN*	2.1	3.8 × 10^−2^	joining chain of multimeric IgA and IgM [Source:HGNC Symbol;Acc:HGNC:5713]
**Downregulated genes**				
1	*ABALON*	−2.1	8.6 × 10^−4^	apoptotic BCL2L1-antisense long noncoding RNA [Source:HGNC Symbol;Acc:HGNC:49667]
2	*AHSP*	−2.3	6.1 × 10^−3^	alpha haemoglobin stabilizing protein [Source:HGNC Symbol;Acc:HGNC:18075]
3	*ALAS2*	−2.2	1.6 × 10^−2^	5’-aminolevulinate synthase 2 [Source:HGNC Symbol;Acc:HGNC:397]
4	*BCL2L1*	−2.0	1.0 × 10^−3^	BCL2 like 1 [Source:HGNC Symbol;Acc:HGNC:992]
5	*CA1*	−2.4	3.6 × 10^−2^	carbonic anhydrase 1 [Source:HGNC Symbol;Acc:HGNC:1368]
6	*EPB42*	−2.2	3.5 × 10^−4^	erythrocyte membrane protein band 4.2 [Source:HGNC Symbol;Acc:HGNC:3381]
7	*HBD*	−2.0	1.7 × 10^−3^	haemoglobin subunit delta [Source:HGNC Symbol;Acc:HGNC:4829]
8	*HBM*	−2.3	1.6 × 10^−2^	haemoglobin subunit mu [Source:HGNC Symbol;Acc:HGNC:4826]
9	*IFIT1B*	−2.2	1.5 × 10^−3^	interferon induced protein with tetratricopeptide repeats 1B [Source:HGNC Symbol;Acc:HGNC:23442]
10	*KLC3*	−2.2	1.8 × 10^−3^	kinesin light chain 3 [Source:HGNC Symbol;Acc:HGNC:20717]
11	*KLF1*	−2.4	2.4 × 10^−4^	Kruppel-like factor 1 [Source:HGNC Symbol;Acc:HGNC:6345]
12	*KRT1*	−2.9	5.8 × 10^−3^	keratin 1 [Source:HGNC Symbol;Acc:HGNC:6412]
13	*MRC2*	−2.2	3.6 × 10^−4^	mannose receptor C type 2 [Source:HGNC Symbol;Acc:HGNC:16875]
14	*MYL4*	−2.2	3.6 × 10^−4^	myosin light chain 4 [Source:HGNC Symbol;Acc:HGNC:7585]
15	*NEAT1_3*	−2.7	4.4 × 10^−2^	Nuclear enriched abundant transcript 1 conserved region 3 [Source:RFAM;Acc:RF01957]
16	*OSBP2*	−2.2	7.1 × 10^−4^	oxysterol binding protein 2 [Source:HGNC Symbol;Acc:HGNC:8504]
17	*PDZK1IP1*	−2.6	2.8 × 10^−6^	PDZK1 interacting protein 1 [Source:HGNC Symbol;Acc:HGNC:16887]
18	*PLVAP*	−2.6	7.3 × 10^−4^	plasmalemma vesicle associated protein [Source:HGNC Symbol;Acc:HGNC:13635]
19	*RUNDC3A*	−2.2	2.1 × 10^−4^	RUN domain containing 3A [Source:HGNC Symbol;Acc:HGNC:16984]
20	*SELENBP1*	−2.2	2.3 × 10^−3^	selenium binding protein 1 [Source:HGNC Symbol;Acc:HGNC:10719]
21	*SHISA4*	−2.2	1.9 × 10^−2^	shisa family member 4 [Source:HGNC Symbol;Acc:HGNC:27139]
22	*SLC4A1*	−2.1	1.8 × 10^−3^	solute carrier family 4 member 1 (Diego blood group) [Source:HGNC Symbol;Acc:HGNC:11027]
23	*SNCA*	−2.2	4.7 × 10^−3^	synuclein alpha [Source:HGNC Symbol;Acc:HGNC:11138]
24	*TNS1*	−2.1	1.4 × 10^−3^	tensin 1 [Source:HGNC Symbol;Acc:HGNC:11973]
25	*TRIM58*	−2.0	6.9 × 10^−3^	tripartite motif containing 58 [Source:HGNC Symbol;Acc:HGNC:24150]
26	*VWCE*	−2.4	8.1 × 10^−4^	von Willebrand factor C and EGF domains [Source:HGNC Symbol;Acc:HGNC:26487]

FC = Fold change between smokers and nonsmokers; a negative sign indicates overexpression in nonsmokers. * corrected *p*-value.

## Data Availability

The data presented in this study are available on request from the corresponding author. The data are not publicly available due to ethical restrictions.

## Supplementary Materials

The supporting information can be downloaded at: https://www.mdpi.com/article/10.3390/ijms241813920/s1.

## Abbreviations

CAD	Coronary artery disease
CHD	Coronary heart disease
CX	Circumflex artery
DEGs	Differentially expressed genes
FC	Fold change
FDR	False discovery rate
GO	Gene Ontology
Igs	Immunoglobulins
LAD	Left anterior descending artery
LM	Left main coronary artery
ND	Nicotine dependence
NGS	Next-generation sequencing
OS	Oxidative stress
RCA	Right coronary artery
RIN	RNA integrity number
SD	Standard deviation

## References

[1] CDC Coronary Artery Disease (CAD). Coronary Artery Disease, This Process Is Called Atherosclerosis. https://www.cdc.gov/heartdisease/coronary_ad.htm#:~:text=Print-.

[2] Libby P., Theroux P. (2005). Pathophysiology of Coronary Artery Disease. Circulation.

[3] Malakar A.K., Choudhury D., Halder B., Paul P., Uddin A., Chakraborty S. (2019). A Review on Coronary Artery Disease, Its Risk Factors, and Therapeutics. J. Cell. Physiol..

[4] Kamceva G., Arsova-Sarafinovska Z., Ruskovska T., Zdravkovska M., Kamceva-Panova L., Stikova E. (2016). Cigarette Smoking and Oxidative Stress in Patients with Coronary Artery Disease. Open Access Maced. J. Med. Sci..

[5] Caliri A.W., Tommasi S., Besaratinia A. (2021). Relationships among Smoking, Oxidative Stress, Inflammation, Macromolecular Damage, and Cancer. Mutat. Res.—Rev. Mutat. Res..

[6] Amit V.K., Sekar K. (2017). Genetics of Coronary Artery Disease: Discovery, Biology and Clinical Translation. Nat. Rev. Genet..

[7] Ding N., Sang Y., Chen J., Ballew S.H., Kalbaugh C.A., Salameh M.J., Blaha M.J., Allison M., Heiss G., Selvin E. (2019). Cigarette Smoking, Smoking Cessation, and Long-Term Risk of 3 Major Atherosclerotic Diseases. J. Am. Coll. Cardiol..

[8] McEvoy J.W., Blaha M.J., DeFilippis A.P., Lima J.A., Bluemke D.A., Hundley W.G., Min J.K., Shaw L.J., Lloyd-Jones D.M., Barr R.G. (2015). Cigarette Smoking and Cardiovascular Events: Role of Inflammation and Subclinical Atherosclerosis: The Multi-Ethnic Study of Atherosclerosis (MESA). Arter. Thromb. Vasc. Biol..

[9] OECD (2021). European Observatory on Health Systems and Policies. State of Health in the EU—Hungary: Country Health Profile.

[10] Nasr N., Soltész B., Sándor J., Ádány R., Fiatal S. (2023). Comparison of Genetic Susceptibility to Coronary Heart Disease in the Hungarian Populations: Risk Prediction Models for Coronary Heart Disease. Genes.

[11] Porsch F., Mallat Z., Binder C.J. (2021). Humoral Immunity in Atherosclerosis and Myocardial Infarction: From B Cells to Antibodies. Cardiovasc. Res..

[12] Hafiz M., Abdullah N., Othman Z., Mohd H., Suri S., Khairuddin A., Yusof M., Jamal R., Rashid A., Rahman A. (2012). Peripheral Blood Gene Expression Profile of Atherosclerotic Coronary Artery Disease in Patients of Different Ethnicity in Malaysia. J. Cardiol..

[13] Li J., Liu S., Cao G., Sun Y., Chen W., Dong F., Xu J., Zhang C., Zhang W. (2018). Nicotine Induces Endothelial Dysfunction and Promotes Atherosclerosis via GTPCH1. J. Cell. Mol. Med..

[14] Banks E., Joshy G., Korda R.J., Stavreski B., Soga K., Egger S., Day C., Clarke N.E., Lewington S., Lopez A.D. (2019). Tobacco Smoking and Risk of 36 Cardiovascular Disease Subtypes: Fatal and Non-Fatal Outcomes in a Large Prospective Australian Study. BMC Med..

[15] Khoramdad M., Leila A.V. (2019). Association between Passive Smoking and Cardiovascular Disease: A Systematic Review and Meta-Analysis. Int. Union Biochem. Mol. Biol..

[16] Salehi N., Janjani P., Tadbiri H., Rozbahani M., Jalilian M. (2021). Effect of Cigarette Smoking on Coronary Arteries and Pattern and Severity of Coronary Artery Disease: A Review. J. Int. Med. Res..

[17] Tolstrup J.S., Hvidtfeldt U.A., Flachs E.M., Spiegelman D., Heitmann B.L., Bälter K., Goldbourt U., Hallmans G., Knekt P., Liu S. (2014). Smoking and Risk of Coronary Heart Disease in Younger, Middle-Aged, and Older Adults. Am. J. Public Health.

[18] Tian X., Chen S., Zuo Y., Zhang Y., Zhang X., Xu Q., Luo Y., Wu S., Wang A. (2022). Association of Lipid, Inflammatory, and Metabolic Biomarkers with Age at Onset for Incident Cardiovascular Disease. BMC Med..

[19] Dugani S.B., Moorthy M.V., Li C., Demler O.V., Alsheikh-Ali A.A., Ridker P.M., Glynn R.J., Mora S. (2021). Association of Lipid, Inflammatory, and Metabolic Biomarkers with Age at Onset for Incident Coronary Heart Disease in Women. JAMA Cardiol..

[20] Jamee A., Abed Y., Jalambo M.O. (2013). Gender Difference and Characteristics Attributed to Coronary Artery Disease in Gaza-Palestine. Glob. J. Health Sci..

[21] Sayed A.I. (2022). Gender Differences in Coronary Artery Disease, Clinical Characteristics, and Angiographic Features in the Jazan Region, Saudi Arabia. Cureus.

[22] Schroeder H.W.J., Cavacini L. (2010). Structure and Function of Immunoglobulins. J. Allergy Clin. Immunol..

[23] Tarbiah N., Todd I., Tighe P.J., Fairclough L.C. (2019). Cigarette Smoking Differentially Affects Immunoglobulin Class Levels in Serum and Saliva: An Investigation and Review. Basic Clin. Pharmacol. Toxicol..

[24] Núñez E., Fuster V., Gómez-Serrano M., Valdivielso J.M., Fernández-Alvira J.M., Martínez-López D., Rodríguez J.M., Bonzon-Kulichenko E., Calvo E., Alfayate A. (2022). Unbiased Plasma Proteomics Discovery of Biomarkers for Improved Detection of Subclinical Atherosclerosis. eBioMedicine.

[25] Piaggeschi G., Rolla S., Rossi N., Brusa D., Naccarati A., Couvreur S., Spector T.D., Roederer M., Mangino M., Cordero F. (2021). Immune Trait Shifts in Association with Tobacco Smoking: A Study in Healthy Women. Front. Immunol..

[26] Ewald D.A., Malajian D., Krueger J.G., Workman C.T., Wang T., Tian S., Litman T., Guttman-yassky E., Suárez-fariñas M. (2015). Meta-Analysis Derived Atopic Dermatitis (MADAD) Transcriptome Defines a Robust AD Signature Highlighting the Involvement of Atherosclerosis and Lipid Metabolism Pathways. BMC Med. Genom..

[27] Wang J.-F., Huang Y., Lu S.-F., Hong H., Xu S.-J., Xie J.-S., Wu Z.-Y., Tang Y., Xu H.-X., Fu S.-P. (2020). Comparative Study of Gene Expression Profiles Rooted in Acute Myocardial Infarction and Ischemic/Reperfusion Rat Models. Am. J. Cardiovasc. Dis..

[28] Vink J.M., Jansen R., Brooks A., Willemsen G., van Grootheest G., de Geus E., Smit J.H., Penninx B.W., Boomsma D.I. (2017). Differential Gene Expression Patterns between Smokers and Non-Smokers: Cause or Consequence?. Addict. Biol..

[29] Haase T., Müller C., Stoffers B., Kirn P., Waldenberger M., Kaiser F.J., Karakas M., Kim S.V., Voss S., Wild P.S. (2023). G Protein-Coupled Receptor 15 Expression Is Associated with Myocardial Infarction. Int. J. Mol. Sci..

[30] Andersen A.M., Lei M.K., Beach S.R.H., Philibert R.A., Sinha S., Colgan J.D. (2020). Cigarette and Cannabis Smoking Effects on GPR15+ Helper T Cell Levels in Peripheral Blood: Relationships with Epigenetic Biomarkers. Genes.

[31] Grievink H.W., Smit V., Huisman B.W., Gal P., Yavuz Y., Klerks C., Binder C.J., Bot I., Kuiper J., Foks A.C. (2022). Cardiovascular Risk Factors: The Effects of Ageing and Smoking on the Immune System, an Observational Clinical Study. Front. Immunol..

[32] Wang C., Song C., Liu Q., Zhang R., Fu R., Wang H., Yin D., Song W., Zhang H., Dou K. (2021). Gene Expression Analysis Suggests Immunological Changes of Peripheral Blood Monocytes in the Progression of Patients with Coronary Artery Disease. Front. Genet..

[33] Ma C., Lu T., He Y., Guo D., Duan L., Jia R., Cai D., Gao T., Chen Z., Xue B. (2023). Comprehensive Analysis of Autophagy-Related Gene Expression Profiles Identified Five Gene Biomarkers Associated with Immune Infiltration and Advanced Plaques in Carotid Atherosclerosis. Orphanet J. Rare Dis..

[34] Xia Y., Brewer A., Bell J.T. (2021). DNA Methylation Signatures of Incident Coronary Heart Disease: Findings from Epigenome-Wide Association Studies. Clin. Epigenetics.

[35] Scott D., Palmer R. (2002). The Influence of Tobacco Smoking on Adhesion Molecule Profiles. Tob. Induc. Dis..

[36] Agha G., Mendelson M.M., Ward-Caviness C.K., Joehanes R., Huan T.X., Gondalia R., Salfati E., Brody J.A., Fiorito G., Bressler J. (2019). Blood Leukocyte DNA Methylation Predicts Risk of Future Myocardial Infarction and Coronary Heart Disease. Circulation.

[37] Petsophonsakul P., Burgmaier M., Willems B., Heeneman S., Stadler N., Gremse F., Reith S., Burgmaier K., Kahles F., Marx N. (2022). Nicotine Promotes Vascular Calcification via Intracellular Ca^2+^-Mediated, Nox5-Induced Oxidative Stress, and Extracellular Vesicle Release in Vascular Smooth Muscle Cells. Cardiovasc. Res..

[38] Li Y., Fu W., Geng Z., Song Y., Yang X., He T., Wu J., Wang B. (2022). A Pan-Cancer Analysis of the Oncogenic Role of Ribonucleotide Reductase Subunit M2 in Human Tumors. PeerJ.

[39] Slizhikova D.K., Zinovyeva M.V., Kuzmin D.V., Snezhkov E.V., Shakhparonov M.I., Dmitriev R.I., Antipova N.V., Zavalova L.L., Sverdlov E.D. (2007). Decreased Expression of the Human Immunoglobulin J-Chain Gene in Squamous Cell Cancer and Adenocarcinoma of the Lungs. Mol. Biol..

[40] Marengo-Rowe A.J. (2006). Structure-Function Relations of Human Hemoglobins. Proc. Baylor Univ. Med. Cent..

[41] Thriveni R., Manshi P., Ramesh D.V., Rachel B., Byatnal A., Kempwade P. (2020). Effects of Smoking on Hemoglobin and Erythrocytes Sedimentation Rate and Its Association with ABO Blood Groups. J. Indian Acad. Oral Med. Radiol..

[42] Rietbrock N., Kunkel S., Worner W., Eyer P. (1992). Oxygen-Dissociation Kinetics in the Blood of Smokers and Non-Smokers: Interaction between Oxygen and Carbon Monoxide at the Hemoglobin Molecule. Naunyn-Schmiedebergs Arch. Pharmacol..

[43] Remigante A., Spinelli S., Pusch M., Sarikas A., Morabito R., Marino A., Dossena S. (2022). Role of SLC4 and SLC26 Solute Carriers during Oxidative Stress. Acta Physiol..

[44] Revin V.V., Gromova N.V., Revina E.S., Samonova A.Y., Tychkov A.Y., Bochkareva S.S., Moskovkin A.A., Kuzmenko T.P. (2019). The Influence of Oxidative Stress and Natural Antioxidants on Morphometric Parameters of Red Blood Cells, the Hemoglobin Oxygen Binding Capacity, and the Activity of Antioxidant Enzymes. Biomed. Res. Int..

[45] Cox T.C., Sadlon T.J., Schwarz Q.P., Matthews C.S., Wise P.D., Cox L.L., Bottomley S.S., May B.K. (2004). The Major Splice Variant of Human 5-Aminolevulinate Synthase-2 Contributes Significantly to Erythroid Heme Biosynthesis. Int. J. Biochem. Cell Biol..

[46] Guauque-Olarte S., Gaudreault N., Piché M.È., Fournier D., Mauriège P., Mathieu P., Bossé Y. (2011). The Transcriptome of Human Epicardial, Mediastinal and Subcutaneous Adipose Tissues in Men with Coronary Artery Disease. PLoS ONE.

[47] Beccacece L., Abondio P., Bini C., Pelotti S. (2023). The Link between Prostanoids and Cardiovascular Diseases. Int. J. Mol. Sci..

[48] Verdugo R.A., Zeller T., Rotival M., Wild P.S., Munzel T., Lackner K.J., Weidmann H., Ninio E., Tregouet D.-A., Cambien F. (2013). Graphical Modeling of Gene Expression in Monocytes Suggests Molecular Mechanisms Explaining Increased Atherosclerosis in Smokers. PLoS ONE.

[49] Benowitz N.L., Jacob P. (1994). Metabolism of Nicotine to Cotinine Studied by a Dual Stable Isotope Method. Clin. Pharmacol. Ther..

[50] Heatherton T.F., Kozlowski L.T., Frecker R.C., Fagerström K.O. (1991). The Fagerström Test for Nicotine Dependence: A Revision of the Fagerström Tolerance Questionnaire. Br. J. Addict..

[51] Sample Size for Microarray Experiments. https://bioinformatics.mdanderson.org/MicroarraySampleSize/.

[52] Bioanalyzer (2020). Agilent 2100 Bioanalyzer System: 2100 Expert Software User’s Guide.

[53] Hukkanen J., Jacob P., Benowitz N.L. (2005). Metabolism and Disposition Kinetics of Nicotine. Pharmacol. Rev..

[54] Garcia-Garcia H.M., Eugène P., McFadden A.F., Mehran R., Stone G.W., Spertus J., Onuma Y., Morel M., van Es G.-A., Zuckerman B. (2018). Standardized End Point Definitions for Coronary Intervention Trials. Circulation.

[55] Andrews S. (2010). FastQC: A Quality Control Tool for High Throughput Sequence Data.

