# Supplementary material for: A Transcriptomic Analysis of Smoking-Induced Gene Expression Alterations in Coronary Artery Disease Patients
Source: Int J Mol Sci. 2023 Sep 10;24(18):13920. doi: 10.3390/ijms241813920 (PMC10530857; doi:10.3390/ijms241813920)
Supplement: Supplementary file 1 [file ijms-24-13920-s001.zip › ijms-2552146-supplementary.pdf]

Supplementary Materials for:

A Transcriptomic Analysis of Smoking-Induced Gene Expression Alterations in Coronary Artery Disease Patients

Mohammed Merzah [1], Szilárd Póliska [2], László Balogh [3], János Sándor [1,4], István Szász [1,4], Shewaye Natae [1] and Szilvia Fiatal [1,*]

[1] Department of Public Health and Epidemiology, Faculty of Medicine, University of Debrecen, H-4032 Debrecen, Hungary; mohammed.merzah@med.unideb.hu (M.M.); sandor.janos@med.unideb.hu (J.S.); natae.shewaye@med.unideb.hu (S.N.)
[2] Department of Biochemistry and Molecular Biology, Faculty of Medicine, University of Debrecen, H-4032 Debrecen, Hungary
[3] Cardiology and Cardiac Surgery Clinic, University of Debrecen, H-4032 Debrecen, Hungary
[4] ELKH-DE Public Health Research Group, Department of Public Health and Epidemiology, Faculty of Medicine, University of Debrecen, H-4032 Debrecen, Hungary
* Correspondence: fiatal.szilvia@med.unideb.hu

Tables of contents:

Table S1: Differentially expressed genes with a fold change of >1.5 (N=61) 2
Table S2: Demographic characteristics of study participants (N=44). 4
Table S3: Differentially expressed genes with a fold change of >1.5 (N=44) 5
Table S4: Gene ontology of the 15 upregulated genes (N=44) 9
Table S5: Gene ontology of the 105 downregulated genes (N=44) 10
Table S6: KEEG pathway of the DEGs (FC>1.5, N=44) 11
Table S7: Differentially expressed genes among smokers based on Nicotine Dependence (N=31) 12

Tables of figures:

Figure S1: The heatmap of the DEGs with a fold change of >1.5 (N=44) 3
Figure S2: The heatmap of the DEGs among smokers based on Nicotine Dependence (n=31)........ 14
Figure S3: GO analysis of the DEGs among smokers categorized based on Nicotine Dependence 15

Table S1. Differentially expressed genes with a fold change of >1.5 (N=61).

No.	Gene Symbol	FC	p-value	Description
**Upregulated genes**				
1	GPR15	6.0	5.4E-12	G protein-coupled receptor 15 [Source:HGNC Symbol;Acc:HGNC:4469]
2	IFI44	1.8	1.8E-02	interferon induced protein 44 [Source:HGNC Symbol;Acc:HGNC:16938]
3	IGKV3-11	1.6	1.6E-02	immunoglobulin kappa variable 3-11 [Source:HGNC Symbol;Acc:HGNC:5815]
4	IGLV1-47	1.5	2.3E-02	immunoglobulin lambda variable 1-47 [Source:HGNC Symbol;Acc:HGNC:5880]
5	IGLV2-23	1.6	4.9E-02	immunoglobulin lambda variable 2-23 [Source:HGNC Symbol;Acc:HGNC:5890]
6	IGLV3-21	1.7	3.2E-02	immunoglobulin lambda variable 3-21 [Source:HGNC Symbol;Acc:HGNC:5905]
**Downregulated genes**				
1	CHI3L1	-1.5	4.5E-02	chitinase 3 like 1 [Source:HGNC Symbol;Acc:HGNC:1932]
2	EPB42	-1.5	3.9E-02	erythrocyte membrane protein band 4.2 [Source:HGNC Symbol;Acc:HGNC:3381]
3	HBQ1	-1.5	4.7E-02	hemoglobin subunit theta 1 [Source:HGNC Symbol;Acc:HGNC:4833]
4	KLF1	-1.6	1.9E-02	Kruppel like factor 1 [Source:HGNC Symbol;Acc:HGNC:6345]
5	PDZK1IP1	-1.7	3.5E-03	PDZK1 interacting protein 1 [Source:HGNC Symbol;Acc:HGNC:16887]
6	PTGDS	-1.6	1.5E-02	prostaglandin D2 synthase [Source:HGNC Symbol;Acc:HGNC:9592]
7	RETN	-1.5	3.6E-02	resistin [Source:HGNC Symbol;Acc:HGNC:20389]

FC= Fold change between smokers and nonsmokers (reference).

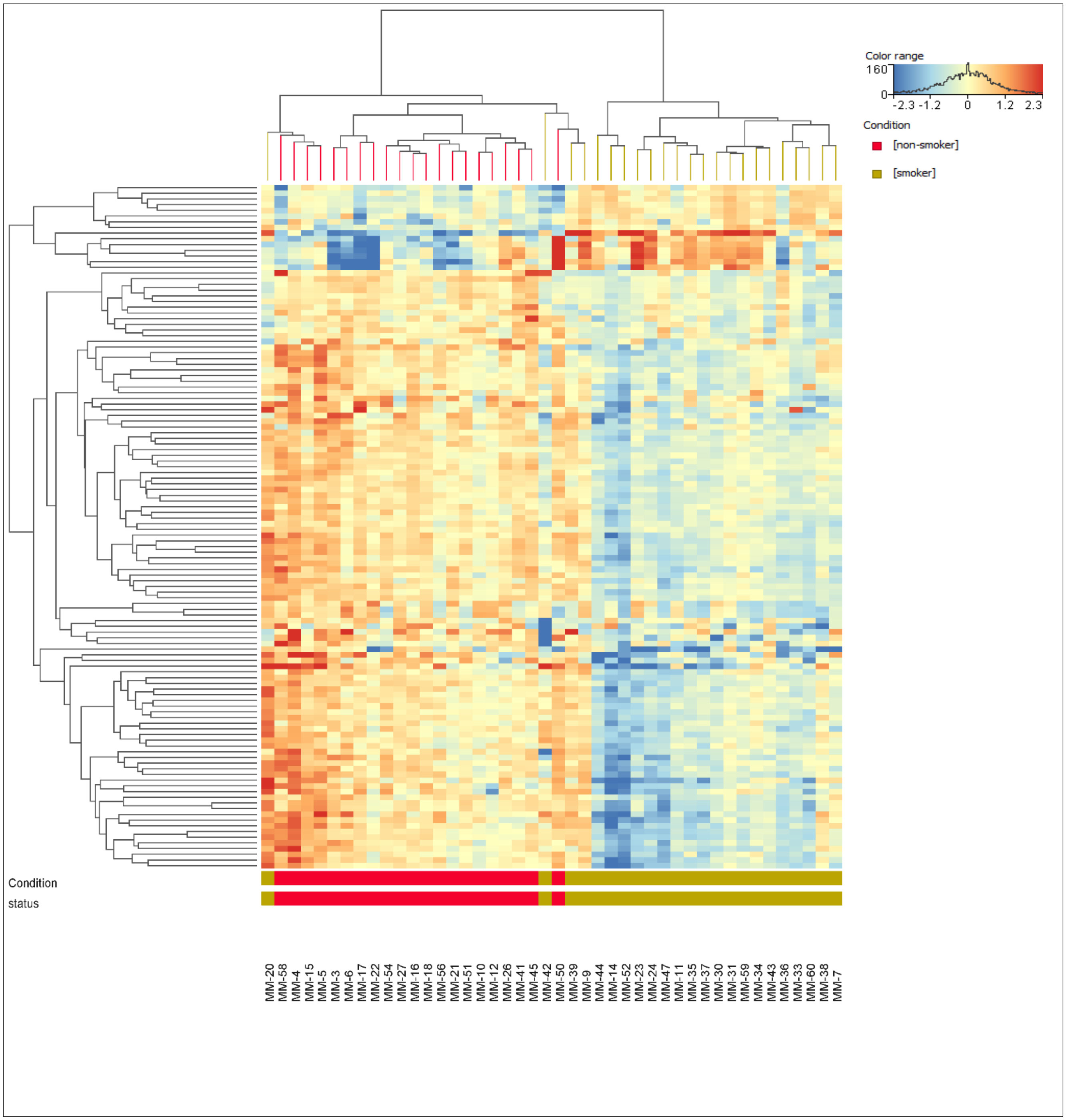

Figure S1. The heatmap of the DEGs with a fold change of >1.5 (N=44).

Table S2. Demographic characteristics of study participants (N=44).

Variables	Total	Smoking status		P-value
		Smokers	Non-smokers	
Sex, n (%)				
**Male**	**18 (63.6)**	**18 (81.8)**	**10 (45.5)**	**<0.001**
**Female**	**16 (36.4)**	**4 (18.2)**	**12 (54.5)**	
**Age, median (range) years**	**68 (32-88)**	**61 (32-75)**	**75 (54-88)**	**<0.001**
Cholesterol (mmol/L), mean (SD)	5.0 (1.4)	5.3 (1.3)	4.7 (1.4)	NS
Triglyceride (mmol/L), mean (SD)	1.5 (0.6)	1.5 (0.5)	1.5 (0.7)	NS
HDL-C (mmol/L), mean (SD)	1.3 (0.6)	1.4 (0.8)	1.2 (0.2)	NS
LDL-C (mmol/L), mean (SD)	3.0 (1.2)	3.1 (1.2)	2.8 (1.3)	NS
**Family history of CAD, n (%)**	**12 (27.3)**	**10 (22.7)**	**2 (9.1)**	**0.02**
Anticoagulation medication, n (%)	44 (100)	22 (100)	22 (100)	NS
BMI, mean (SD)	26.3 (3.0)	25.6 (3.1)	27.0 (2.8)	NS
Blood pressure (mmHg), mean (SD)				
Systolic	126.1 (22.5)	120.6 (23.9)	131.6 (20.1)	NS
Diastolic	72.8 (11.4)	71.5 (13.3)	74.1 (9.4)	NS
Plasma cotinine ng/mL, mean (SD)	2.8 (1.6)	3.0 (1.5)	2.5(1.7)	NS
Angiography findings, n (%)				
>2 arteries stenosis	36 (81.8)	18 (81.8)	18 (81.8)	NS
Number of affected arteries, median (range)	2 (1-3)	2 (1-3)	2 (1-3)	NS
LAD	37 (84.1)	18 (81.8)	19 (86.4)	NS
LM	7 (15.9)	5 (22.7)	2 (9.1)	NS
CX	26 (59.1)	11 (50.0)	15 (68.2)	NS
RCA	26 (59.1)	14 (63.6)	12 (54.5)	NS

n = number of subject; NS= Non-significant; SD= Standard deviation. LAD= Left anterior descending artery; LM= Left main coronary artery; CX= Circumflex artery; RCA= Right coronary artery; Bold text identifies significant differences between study groups.

Table S3. Differentially expressed genes with a fold change of >1.5 (N=44).

No.	Gene Symbol	FC	p-value	p-value*	Description
	**Upregulated genes**				
1	CCR7	1.6	3.5E-03	3.1E-02	C-C motif chemokine receptor 7 [Source:HGNC Symbol;Acc:HGNC:1608]
2	CD28	1.6	4.5E-05	2.6E-03	CD28 molecule [Source:HGNC Symbol;Acc:HGNC:1653]
3	GPR15	7.5	1.8E-13	1.4E-09	G protein-coupled receptor 15 [Source:HGNC Symbol;Acc:HGNC:4469]
4	IGHA2	2.6	2.8E-03	2.8E-02	immunoglobulin heavy constant alpha 2 (A2m marker) [Source:HGNC Symbol;Acc:HGNC:5479]
5	IGKV3-11	2.2	2.2E-04	6.2E-03	immunoglobulin kappa variable 3-11 [Source:HGNC Symbol;Acc:HGNC:5815]
6	IGLC1	2.0	6.8E-03	4.5E-02	immunoglobulin lambda constant 1 [Source:HGNC Symbol;Acc:HGNC:5855]
7	IGLL5	1.9	7.3E-03	4.6E-02	immunoglobulin lambda like polypeptide 5 [Source:HGNC Symbol;Acc:HGNC:38476]
8	IGLV3-21	2.4	3.4E-03	3.0E-02	immunoglobulin lambda variable 3-21 [Source:HGNC Symbol;Acc:HGNC:5905]
9	ITGA6	1.5	5.5E-06	7.7E-04	integrin subunit alpha 6 [Source:HGNC Symbol;Acc:HGNC:6142]
10	JCHAIN	2.1	4.9E-03	3.8E-02	joining chain of multimeric IgA and IgM [Source:HGNC Symbol;Acc:HGNC:5713]
11	LEF1	1.7	2.1E-05	1.6E-03	lymphoid enhancer binding factor 1 [Source:HGNC Symbol;Acc:HGNC:6551]
12	RCAN3	1.5	3.6E-05	2.2E-03	RCAN family member 3 [Source:HGNC Symbol;Acc:HGNC:3042]
13	TRAT1	1.6	4.8E-04	1.0E-02	T cell receptor associated transmembrane adaptor 1 [Source:HGNC Symbol;Acc:HGNC:30698]
14	TRAV13-1	1.5	0.0003	0.0081	T cell receptor alpha variable 13-1 [Source:HGNC Symbol;Acc:HGNC:12108]
15	TRAV4	1.6	4.6E-03	3.6E-02	T cell receptor alpha variable 4 [Source:HGNC Symbol;Acc:HGNC:12140]
	**Downregulated genes**				
1	ABALON	-2.1	6.9E-06	8.6E-04	apoptotic BCL2L1-antisense long non-coding RNA [Source:HGNC Symbol;Acc:HGNC:49667]
2	ADIPOR1	-1.5	1.9E-04	5.8E-03	adiponectin receptor 1 [Source:HGNC Symbol;Acc:HGNC:24040]
3	AHSP	-2.3	2.1E-04	6.1E-03	alpha hemoglobin stabilizing protein [Source:HGNC Symbol;Acc:HGNC:18075]
4	ALAS2	-2.2	1.1E-03	1.6E-02	5'-aminolevulinate synthase 2 [Source:HGNC Symbol;Acc:HGNC:397]
5	ANK1	-1.9	3.2E-05	2.0E-03	ankyrin 1 [Source:HGNC Symbol;Acc:HGNC:492]
6	ARHGEF40	-1.5	1.7E-04	5.4E-03	Rho guanine nucleotide exchange factor 40 [Source:HGNC Symbol;Acc:HGNC:25516]
7	ASCC2	-1.7	2.3E-08	2.6E-05	activating signal cointegrator 1 complex subunit 2 [Source:HGNC Symbol;Acc:HGNC:24103]
8	BAG1	-1.8	1.0E-08	1.3E-05	BCL2 associated athanogene 1 [Source:HGNC Symbol;Acc:HGNC:937]
9	BCL2L1	-2.0	9.3E-06	1.0E-03	BCL2 like 1 [Source:HGNC Symbol;Acc:HGNC:992]
10	BLVRB	-1.8	3.6E-09	7.0E-06	biliverdin reductase B [Source:HGNC Symbol;Acc:HGNC:1063]
11	CA1	-2.4	4.6E-03	3.6E-02	carbonic anhydrase 1 [Source:HGNC Symbol;Acc:HGNC:1368]
12	CARM1	-1.6	2.2E-05	1.7E-03	coactivator associated arginine methyltransferase 1 [Source:HGNC Symbol;Acc:HGNC:23393]
13	CYSTM1	-1.6	1.1E-03	1.6E-02	cysteine rich transmembrane module containing 1 [Source:HGNC Symbol;Acc:HGNC:30239]
14	DCAF12	-1.6	7.1E-05	3.3E-03	DDB1 and CUL4 associated factor 12 [Source:HGNC Symbol;Acc:HGNC:19911]
15	DMTN	-1.9	2.3E-05	1.7E-03	dematin actin binding protein [Source:HGNC Symbol;Acc:HGNC:3382]
16	DPM2	-1.8	8.5E-08	5.9E-05	dolichyl-phosphate mannosyltransferase subunit 2, regulatory [Source:HGNC Symbol;Acc:HGNC:3006]
17	DUSP1	-1.6	6.6E-06	8.3E-04	dual specificity phosphatase 1 [Source:HGNC Symbol;Acc:HGNC:3064]
18	EPB42	-2.2	1.5E-06	3.5E-04	erythrocyte membrane protein band 4.2 [Source:HGNC Symbol;Acc:HGNC:3381]
19	FAM210B	-1.6	7.2E-04	1.2E-02	family with sequence similarity 210 member B [Source:HGNC Symbol;Acc:HGNC:16102]
20	FAXDC2	-1.9	9.0E-06	1.0E-03	fatty acid hydroxylase domain containing 2 [Source:HGNC Symbol;Acc:HGNC:1334]

21	FBXO7	-1.9	3.4E-07	1.8E-04	F-box protein 7 [Source:HGNC Symbol;Acc:HGNC:13586]
22	FKBP8	-1.7	2.6E-05	1.8E-03	FK506 binding protein 8 [Source:HGNC Symbol;Acc:HGNC:3724]
23	FUNDC2P1	-1.8	4.8E-06	7.3E-04	FUN14 domain containing 2 pseudogene 1 [Source:HGNC Symbol;Acc:HGNC:17253]
24	GATA1	-1.8	5.1E-07	1.8E-04	GATA binding protein 1 [Source:HGNC Symbol;Acc:HGNC:4170]
25	GLRX5	-1.6	1.9E-03	2.1E-02	glutaredoxin 5 [Source:HGNC Symbol;Acc:HGNC:20134]
26	GMPR	-1.8	2.4E-04	6.6E-03	guanosine monophosphate reductase [Source:HGNC Symbol;Acc:HGNC:4376]
27	GPR146	-1.6	1.8E-03	2.1E-02	G protein-coupled receptor 146 [Source:HGNC Symbol;Acc:HGNC:21718]
28	GPX1	-1.6	2.2E-05	1.7E-03	glutathione peroxidase 1 [Source:HGNC Symbol;Acc:HGNC:4553]
29	GPX1P1	-1.6	2.1E-05	1.6E-03	glutathione peroxidase pseudogene 1 [Source:HGNC Symbol;Acc:HGNC:4560]
30	GUK1	-1.6	1.5E-05	1.4E-03	guanylate kinase 1 [Source:HGNC Symbol;Acc:HGNC:4693]
31	GYPC	-1.8	2.6E-06	5.2E-04	glycophorin C (Gerbich blood group) [Source:HGNC Symbol;Acc:HGNC:4704]
32	HAGH	-1.6	5.7E-06	7.8E-04	hydroxyacylglutathione hydrolase [Source:HGNC Symbol;Acc:HGNC:4805]
33	HBA1	-2.0	4.2E-07	1.8E-04	hemoglobin subunit alpha 1 [Source:HGNC Symbol;Acc:HGNC:4823]
34	HBA2	-1.9	8.6E-07	2.4E-04	hemoglobin subunit alpha 2 [Source:HGNC Symbol;Acc:HGNC:4824]
35	HBB	-2.0	2.5E-05	1.8E-03	hemoglobin subunit beta [Source:HGNC Symbol;Acc:HGNC:4827]
36	HBD	-2.0	2.4E-05	1.7E-03	hemoglobin subunit delta [Source:HGNC Symbol;Acc:HGNC:4829]
37	HBM	-2.3	1.1E-03	1.6E-02	hemoglobin subunit mu [Source:HGNC Symbol;Acc:HGNC:4826]
38	HBQ1	-1.8	2.6E-03	2.6E-02	hemoglobin subunit theta 1 [Source:HGNC Symbol;Acc:HGNC:4833]
39	HMBS	-1.5	3.2E-05	2.0E-03	hydroxymethylbilane synthase [Source:HGNC Symbol;Acc:HGNC:4982]
40	HOTAIRM1_4	-1.6	2.4E-03	2.5E-02	HOX antisense intergenic RNA myeloid 1 conserved region 4 [Source:RFAM;Acc:RF01978]
41	IFIT1B	-2.2	1.7E-05	1.5E-03	interferon induced protein with tetratricopeptide repeats 1B [Source:HGNC Symbol;Acc:HGNC:23442]
42	IL1R2	-1.6	3.5E-03	3.0E-02	interleukin 1 receptor type 2 [Source:HGNC Symbol;Acc:HGNC:5994]
43	JHDM1D-AS1	-1.5	3.7E-05	2.2E-03	JHDM1D antisense RNA 1 (head to head) [Source:HGNC Symbol;Acc:HGNC:48959]
44	KLC3	-2.2	2.5E-05	1.8E-03	kinesin light chain 3 [Source:HGNC Symbol;Acc:HGNC:20717]
45	KLF1	-2.4	8.7E-07	2.4E-04	Kruppel like factor 1 [Source:HGNC Symbol;Acc:HGNC:6345]
46	KRT1	-2.9	1.9E-04	5.8E-03	keratin 1 [Source:HGNC Symbol;Acc:HGNC:6412]
47	LINC00694	-1.5	1.4E-03	1.8E-02	long intergenic non-protein coding RNA 694 [Source:HGNC Symbol;Acc:HGNC:44570]
48	LYL1	-1.7	9.1E-08	5.9E-05	LYL1, basic helix-loop-helix family member [Source:HGNC Symbol;Acc:HGNC:6734]
49	MED25	-1.5	3.5E-08	3.3E-05	mediator complex subunit 25 [Source:HGNC Symbol;Acc:HGNC:28845]
50	Metazoa_SRP	-1.7	1.8E-03	2.1E-02	Metazoan signal recognition particle RNA [Source:RFAM;Acc:RF00017]
51	MFSD2B	-1.6	2.9E-05	1.9E-03	major facilitator superfamily domain containing 2B [Source:HGNC Symbol;Acc:HGNC:37207]
52	MIR24-2	-1.7	4.6E-07	1.8E-04	microRNA 24-2 [Source:HGNC Symbol;Acc:HGNC:31608]
53	MRC2	-2.2	1.7E-06	3.6E-04	mannose receptor C type 2 [Source:HGNC Symbol;Acc:HGNC:16875]
54	MYL4	-2.2	1.5E-06	3.6E-04	myosin light chain 4 [Source:HGNC Symbol;Acc:HGNC:7585]
55	NEAT1_3	-2.7	6.7E-03	4.4E-02	Nuclear enriched abundant transcript 1 conserved region 3 [Source:RFAM;Acc:RF01957]
56	NFIX	-1.8	6.3E-07	2.0E-04	nuclear factor I X [Source:HGNC Symbol;Acc:HGNC:7788]
57	NINJ2	-1.5	2.0E-05	1.6E-03	ninjurin 2 [Source:HGNC Symbol;Acc:HGNC:7825]
58	NMUR1	-1.5	6.3E-03	4.3E-02	neuromedin U receptor 1 [Source:HGNC Symbol;Acc:HGNC:4518]
59	NPRL3	-1.6	1.4E-04	4.7E-03	NPR3 like, GATOR1 complex subunit [Source:HGNC Symbol;Acc:HGNC:14124]
60	NT5M	-1.9	2.1E-04	6.1E-03	5',3'-nucleotidase, mitochondrial [Source:HGNC Symbol;Acc:HGNC:15769]

61	OR2W3	-1.8	6.9E-03	4.5E-02	olfactory receptor family 2 subfamily W member 3 [Source:HGNC Symbol;Acc:HGNC:15021]
62	OSBP2	-2.2	4.4E-06	7.1E-04	oxysterol binding protein 2 [Source:HGNC Symbol;Acc:HGNC:8504]
63	PCSK1N	-1.7	2.7E-03	2.7E-02	proprotein convertase subtilisin/kexin type 1 inhibitor [Source:HGNC Symbol;Acc:HGNC:17301]
64	PDZK1IP1	-2.6	1.1E-09	2.8E-06	PDZK1 interacting protein 1 [Source:HGNC Symbol;Acc:HGNC:16887]
65	PHOSPHO1	-1.7	3.9E-07	1.8E-04	phosphoethanolamine/phosphocholine phosphatase [Source:HGNC Symbol;Acc:HGNC:16815]
66	PLEK2	-1.6	1.9E-03	2.1E-02	pleckstrin 2 [Source:HGNC Symbol;Acc:HGNC:19238]
67	PLVAP	-2.6	4.6E-06	7.3E-04	plasmalemma vesicle associated protein [Source:HGNC Symbol;Acc:HGNC:13635]
68	POLL	-1.6	7.0E-08	5.9E-05	DNA polymerase lambda [Source:HGNC Symbol;Acc:HGNC:9184]
69	PTGDS	-1.8	5.7E-03	4.1E-02	prostaglandin D2 synthase [Source:HGNC Symbol;Acc:HGNC:9592]
70	R3HDM4	-1.5	5.2E-07	1.8E-04	R3H domain containing 4 [Source:HGNC Symbol;Acc:HGNC:28270]
71	RBM38	-1.6	1.1E-04	4.2E-03	RNA binding motif protein 38 [Source:HGNC Symbol;Acc:HGNC:15818]
72	RILP	-1.7	1.1E-04	4.1E-03	Rab interacting lysosomal protein [Source:HGNC Symbol;Acc:HGNC:30266]
73	RN7SL1	-1.6	6.3E-03	4.3E-02	RNA, 7SL, cytoplasmic 1 [Source:HGNC Symbol;Acc:HGNC:10038]
74	RN7SL2	-1.6	5.2E-03	3.9E-02	RNA, 7SL, cytoplasmic 2 [Source:HGNC Symbol;Acc:HGNC:23134]
75	RUNDC3A	-2.2	7.2E-07	2.1E-04	RUN domain containing 3A [Source:HGNC Symbol;Acc:HGNC:16984]
76	SELENBP1	-2.2	4.0E-05	2.3E-03	selenium binding protein 1 [Source:HGNC Symbol;Acc:HGNC:10719]
77	SHISA4	-2.2	1.5E-03	1.9E-02	shisa family member 4 [Source:HGNC Symbol;Acc:HGNC:27139]
78	SLC25A39	-1.9	8.4E-06	9.9E-04	solute carrier family 25 member 39 [Source:HGNC Symbol;Acc:HGNC:24279]
79	SLC31A2	-1.5	3.9E-06	6.4E-04	solute carrier family 31 member 2 [Source:HGNC Symbol;Acc:HGNC:11017]
80	SLC38A5	-1.6	4.8E-04	9.9E-03	solute carrier family 38 member 5 [Source:HGNC Symbol;Acc:HGNC:18070]
81	SLC4A1	-2.1	2.6E-05	1.8E-03	solute carrier family 4 member 1 (Diego blood group) [Source:HGNC Symbol;Acc:HGNC:11027]
82	SLC6A8	-1.9	5.7E-05	2.9E-03	solute carrier family 6 member 8 [Source:HGNC Symbol;Acc:HGNC:11055]
83	SMOX	-1.5	1.4E-03	1.8E-02	spermine oxidase [Source:HGNC Symbol;Acc:HGNC:15862]
84	SNCA	-2.2	1.4E-04	4.7E-03	synuclein alpha [Source:HGNC Symbol;Acc:HGNC:11138]
85	SPTB	-1.7	7.5E-04	1.3E-02	spectrin beta, erythrocytic [Source:HGNC Symbol;Acc:HGNC:11274]
86	ST6GALNAC4	-1.5	3.1E-04	7.7E-03	ST6 N-acetylgalactosaminide alpha-2,6-sialyltransferase 4 [Source:HGNC Symbol;Acc:HGNC:17846]
87	STRADB	-1.8	2.9E-04	7.4E-03	STE20-related kinase adaptor beta [Source:HGNC Symbol;Acc:HGNC:13205]
88	TAGLN2	-1.5	3.4E-06	6.0E-04	transgelin 2 [Source:HGNC Symbol;Acc:HGNC:11554]
89	TAGLN2P1	-1.5	3.2E-06	5.9E-04	transgelin 2 pseudogene 1 [Source:HGNC Symbol;Acc:HGNC:21739]
90	TAL1	-1.6	1.0E-04	4.0E-03	TAL bHLH transcription factor 1, erythroid differentiation factor [Source:HGNC Symbol;Acc:HGNC:11556]
91	TESC	-1.8	8.2E-08	5.9E-05	tescalcin [Source:HGNC Symbol;Acc:HGNC:26065]
92	TMCC2	-1.9	5.8E-05	3.0E-03	transmembrane and coiled-coil domain family 2 [Source:HGNC Symbol;Acc:HGNC:24239]
93	TMOD1	-1.5	1.8E-03	2.1E-02	tropomodulin 1 [Source:HGNC Symbol;Acc:HGNC:11871]
94	TNS1	-2.1	1.4E-05	1.4E-03	tensin 1 [Source:HGNC Symbol;Acc:HGNC:11973]
95	TPGS2	-1.5	1.9E-05	1.6E-03	tubulin polyglutamylase complex subunit 2 [Source:HGNC Symbol;Acc:HGNC:24561]
96	TREM1	-1.5	3.7E-06	6.4E-04	triggering receptor expressed on myeloid cells 1 [Source:HGNC Symbol;Acc:HGNC:17760]
97	TRIM58	-2.0	2.6E-04	6.9E-03	tripartite motif containing 58 [Source:HGNC Symbol;Acc:HGNC:24150]
98	TSPAN2	-1.5	2.7E-04	7.0E-03	tetraspanin 2 [Source:HGNC Symbol;Acc:HGNC:20659]
99	TSPAN5	-1.8	4.3E-07	1.8E-04	tetraspanin 5 [Source:HGNC Symbol;Acc:HGNC:17753]

100	UBB	-2.0	3.4E-07	1.8E-04	ubiquitin B [Source:HGNC Symbol;Acc:HGNC:12463]
101	UBBP1	-1.9	1.3E-06	3.2E-04	ubiquitin B pseudogene 1 [Source:HGNC Symbol;Acc:HGNC:12464]
102	UBBP4	-1.9	1.6E-06	3.6E-04	ubiquitin B pseudogene 4 [Source:HGNC Symbol;Acc:HGNC:12467]
103	UBXN6	-1.7	5.1E-06	7.4E-04	UBX domain protein 6 [Source:HGNC Symbol;Acc:HGNC:14928]
104	VWCE	-2.4	6.3E-06	8.1E-04	von Willebrand factor C and EGF domains [Source:HGNC Symbol;Acc:HGNC:26487]
105	YBX3	-1.6	7.8E-04	1.3E-02	Y-box binding protein 3 [Source:HGNC Symbol;Acc:HGNC:2428]

FC = Fold change between smokers and nonsmokers (reference). * corrected p-value.

Table S4. Gene ontology of the 15 upregulated genes (N=44).

Term ID	Term description	Strength	FDR	Matching proteins in our network (labels)
**Biological process**				
GO:0050900	Leukocyte migration	1.54	0.0018	CCR7,IGJ,GPR15,ITGA6,IGLL5
GO:0002376	Immune system process	0.85	0.0035	CCR7,IGJ,LEF1,GPR15,TRAT1,CD28,ITGA6,IGLL5
GO:0016477	Cell migration	1.16	0.0035	CCR7,IGJ,LEF1,GPR15,ITGA6,IGLL5
GO:0022409	Positive regulation of cell-cell adhesion	1.5	0.0099	CCR7,LEF1,CD28,ITGA6
GO:0051251	Positive regulation of lymphocyte activation	1.48	0.0099	CCR7,LEF1,CD28,IGLL5
GO:0002250	Adaptive immune response	1.44	0.012	IGJ,LEF1,TRAT1,IGLL5
GO:0045060	Negative thymic T cell selection	2.6	0.0151	CCR7,CD28
GO:0002863	Positive regulation of inflammatory response to antigenic stimulus	2.56	0.0156	CCR7,CD28
GO:0006955	Immune response	0.91	0.0157	CCR7,IGJ,LEF1,TRAT1,CD28,IGLL5
GO:0002684	Positive regulation of immune system process	1.06	0.0192	CCR7,LEF1,TRAT1,CD28,IGLL5
GO:0030217	T cell differentiation	1.66	0.0192	CCR7,LEF1,CD28
GO:0050862	Positive regulation of T cell receptor signaling pathway	2.46	0.0192	CCR7,TRAT1
GO:0044419	Interspecies interaction between organisms	0.84	0.0292	CCR7,IGJ,LEF1,GPR15,CD28,IGLL5
GO:0051897	Positive regulation of protein kinase B signaling	1.58	0.0292	CCR7,TRAT1,CD28
GO:0032673	Regulation of interleukin-4 production	2.18	0.0372	LEF1,CD28
GO:0050851	Antigen receptor-mediated signaling pathway	1.52	0.0372	TRAT1,CD28,IGLL5
GO:0050870	Positive regulation of T cell activation	1.49	0.0403	CCR7,LEF1,CD28
GO:0007165	Signal transduction	0.55	0.0407	CCR7,LEF1,GPR15,TRAT1,CD28,RCAN3,ITGA6,IGLL5
GO:0050778	Positive regulation of immune response	1.16	0.0407	CCR7,TRAT1,CD28,IGLL5
GO:0050900	Leukocyte migration	1.54	0.0018	
**Cellular Component**				
GO:0009897	External side of plasma membrane	1.42	0.0115	CCR7,CD28,ITGA6,IGLL5
GO:0042571	Immunoglobulin complex, circulating	2.79	0.0115	IGJ,IGLL5
GO:0098636	Protein complex involved in cell adhesion	2.09	0.0422	CD28,ITGA6

GO = Gene ontology; FDR= False discovery rate.

Table S5. Gene ontology of the 105 downregulated genes (N=44).

Term ID	Term description	Strength	FDR	Matching proteins in our network (labels)
**Biological process**				
GO:0030218	Erythrocyte differentiation	1.37	3.03E-06	SLC4A1,KLF1,TAL1,EPB42,AHSP,ALAS2,FAM210B,GATA1,DMTN
GO:0042744	Hydrogen peroxide catabolic process	1.73	3.03E-06	HBQ1,HBA2,HBA1,HBB,HBM,HBD,GPX1
GO:0048821	Erythrocyte development	1.64	3.03E-06	SLC4A1,TAL1,EPB42,ALAS2,FAM210B,GATA1,DMTN
GO:0061515	Myeloid cell development	1.45	3.03E-06	SLC4A1,TAL1,EPB42,ALAS2,TSPAN2,FAM210B,GATA1,DMTN
GO:0030099	Myeloid cell differentiation	0.98	0.00016	SLC4A1,KLF1,TAL1,EPB42,AHSP,ALAS2,TSPAN2,FAM210B,GATA1,DMTN
GO:0098869	Cellular oxidant detoxification	1.22	0.00042	HBQ1,HBA2,HBA1,HBB,HBM,HBD,GPX1
GO:0015701	Bicarbonate transport	1.39	0.0023	HBA2,SLC4A1,HBA1,HBB,CA1
GO:0045639	Positive regulation of myeloid cell differentiation	1.11	0.0071	TAL1,FAXDC2,TESC,TRIM58,FAM210B,GATA1
GO:0002520	Immune system development	0.63	0.0087	SLC4A1,LYL1,KLF1,TAL1,EPB42,AHSP,GLRX5,ALAS2,TSPAN2,POLL,FAM210B,GATA1,DMTN
GO:0030097	Hemopoiesis	0.65	0.0109	SLC4A1,LYL1,KLF1,TAL1,EPB42,AHSP,GLRX5,ALAS2,TSPAN2,FAM210B,GATA1,DMTN
GO:0045648	Positive regulation of erythrocyte differentiation	1.42	0.0144	TAL1,TRIM58,FAM210B,GATA1
GO:0042168	Heme metabolic process	1.41	0.0154	BLVRB,HMBS,ALAS2,SLC25A39
GO:0020027	Hemoglobin metabolic process	1.76	0.0194	EPB42,AHSP,ALAS2
GO:0043249	Erythrocyte maturation	1.63	0.038	TAL1,EPB42,FAM210B
**Molecular function**				
GO:0005344	Oxygen carrier activity	1.96	1.06E-06	HBQ1,HBA2,HBA1,HBB,HBM,HBD
GO:0019825	Oxygen binding	1.55	6.60E-05	HBQ1,HBA2,HBA1,HBB,HBM,HBD
GO:0030492	Hemoglobin binding	1.98	0.00034	SLC4A1,AHSP,HBB,HBD
GO:0043177	Organic acid binding	0.87	0.0129	HBQ1,HBA2,HBA1,ALAS2,HBB,HBM,PTGDS,HBD
GO:0020037	Heme binding	0.98	0.0327	HBQ1,HBA2,HBA1,HBB,HBM,HBD
**Cellular Component**				
GO:0005833	Hemoglobin complex	2.09	3.19E-09	HBQ1,HBA2,AHSP,HBA1,HBB,HBM,HBD
GO:0031838	Haptoglobin-hemoglobin complex	2.06	8.98E-08	HBQ1,HBA2,HBA1,HBB,HBM,HBD
GO:0030863	Cortical cytoskeleton	1.12	0.0047	GYPC,TMOD1,SLC4A1,EPB42,DMTN,SPTB
GO:0014731	Spectrin-associated cytoskeleton	1.85	0.009	ANK1,DMTN,SPTB
GO:0072562	Blood microparticle	1.05	0.009	HBA2,KRT1,SLC4A1,HBA1,HBB,HBD
GO:0005829	Cytosol	0.23	0.0435	STRADB,HBQ1,YBX3,HBA2,KRT1,TMOD1,GMPR,BLVRB,ANK1,FBXO7,HMBS,ARHGEF40,UBXN6,RILP,BCL2L1,UBB,AHSP,HBA1,CARM1,OSBP2,HBB,TESC,SNCA,MYL4,RBM38,HBM,GUK1,TAGLN2,IFIT1B,HBD,HAGH,SELENBP1,PHOSPHO1,GPX1,RUNDC3A,BAG1,DMTN,CA1,SPTB,FKBP8,SMOX
GO:0071682	Endocytic vesicle lumen	1.5	0.0435	HBA2,HBA1,HBB

GO = Gene ontology; FDR= False discovery rate.

Table S6. KEEG pathway of the DEGs (FC>1.5, N=44).

Term ID	Term description	Strength	FDR	Matching proteins in our network (labels)
**Downregulated genes**				
hsa04060	Cytokine-cytokine receptor interaction	1.21	0.014	CCR7,IL7R,CCR4,IL2RA
hsa04061	Viral protein interaction with cytokine and cytokine receptor	1.56	0.014	CCR7,CCR4,IL2RA
hsa04640	Hematopoietic cell lineage	1.58	0.014	IL7R,IL2RA,ITGA6
hsa05200	Pathways in cancer	1.05	0.014	LEF1,IL7R,TCF7,IL2RA,ITGA6
hsa05412	Arrhythmogenic right ventricular cardiomyopathy	1.66	0.014	LEF1,TCF7,ITGA6
hsa05167	Kaposi sarcoma-associated herpesvirus infection	1.27	0.027	LEF1,CCR4,TCF7
hsa05216	Thyroid cancer	1.81	0.027	LEF1,TCF7
hsa05213	Endometrial cancer	1.61	0.049	LEF1,TCF7
**Downregulated genes**				
hsa00860	Porphyrin and chlorophyll metabolism	1.27	0.0301	BLVRB,HMBS,ALAS2,FECH

has = Homo sapiens (human); FDR= False discovery rate.

Table S7. Differentially expressed genes among smokers based on nicotine dependence (N=31).

Gene Symbol	p-value	FC high ND vs. low ND	Regulation high ND vs. low ND	FC low moderate ND vs. low ND	Regulation low moderate ND vs. low ND	FC moderate ND vs. low ND	Regulation moderate ND vs. low ND	Description
ADPGK-AS1	0.03	1.6	up	1.5	up	1.2	up	ADPGK antisense RNA 1 [Source:HGNC Symbol;Acc:HGNC:44144]
ASB16	0.01	1.5	up	1.3	up	1.1	up	ankyrin repeat and SOCS box containing 16 [Source:HGNC Symbol;Acc:HGNC:19768]
HRH4	0.04	1.6	up	-1.5	down	-1.6	down	histamine receptor H4 [Source:HGNC Symbol;Acc:HGNC:17383]
HCG4P5	0.01	1.6	up	-1.1	down	-2.3	down	HLA complex group 4 pseudogene 5 [Source:HGNC Symbol;Acc:HGNC:22925]
IGKV1-12	0.02	9.1	up	1.8	up	4.2	up	immunoglobulin kappa variable 1-12 [Source:HGNC Symbol;Acc:HGNC:5730]
HLA-A	0.01	1.8	up	1.7	up	-2.2	down	major histocompatibility complex, class I, A [Source:HGNC Symbol;Acc:HGNC:4931]
RN7SL2	0.04	2.4	up	2.0	up	1.4	up	RNA, 7SL, cytoplasmic 2 [Source:HGNC Symbol;Acc:HGNC:23134]
SCGB3A1	0.01	30.2	up	3.7	up	1.6	up	secretoglobin family 3A member 1 [Source:HGNC Symbol;Acc:HGNC:18384]
SPTLC1P2	0.03	2.4	up	1.6	up	1.3	up	serine palmitoyltransferase long chain base subunit 1 pseudogene 2 [Source:HGNC Symbol;Acc:HGNC:39669]
SLC29A1	0.03	1.5	up	-1.2	down	-1.7	down	solute carrier family 29 member 1 (Augustine blood group) [Source:HGNC Symbol;Acc:HGNC:11003]
TRAJ33	0.04	1.8	up	-1.9	down	-1.5	down	T cell receptor alpha joining 33 [Source:HGNC Symbol;Acc:HGNC:12063]
UBE2SP2	0.01	1.6	up	2.0	up	1.3	up	ubiquitin conjugating enzyme E2 S pseudogene 2 [Source:HGNC Symbol;Acc:HGNC:32196]
USP9Y	0.04	19.0	up	3.9	up	3.6	up	ubiquitin specific peptidase 9, Y-linked [Source:HGNC Symbol;Acc:HGNC:12633]
ZNF696	0.01	1.9	up	1.1	up	1.4	up	zinc finger protein 696 [Source:HGNC Symbol;Acc:HGNC:25872]
ARMC10P1	0.04	-2.5	down	-1.2	down	1.0	up	armadillo repeat containing 10 pseudogene 1 [Source:HGNC Symbol;Acc:HGNC:43646]
CACYBPP2	0.03	-2.8	down	-1.9	down	-1.6	down	calcyclin binding protein pseudogene 2 [Source:HGNC Symbol;Acc:HGNC:45123]
CCT5P1	0.02	-1.9	down	-1.2	down	-1.4	down	chaperonin containing TCP1 subunit 5 pseudogene 1 [Source:HGNC Symbol;Acc:HGNC:35135]
LYPD2	0.01	-2.0	down	-4.3	down	-8.5	down	LY6/PLAUR domain containing 2 [Source:HGNC Symbol;Acc:HGNC:25215]

MTATP8P2	0.02	-3.1	down	-1.4	down	-1.1	down	mitochondrially encoded ATP synthase 8 pseudogene 2 [Source:HGNC Symbol;Acc:HGNC:44572]
MTCO3P12	0.02	-1.7	down	1.0	up	-1.2	down	mitochondrially encoded cytochrome c oxidase III pseudogene 12 [Source:HGNC Symbol;Acc:HGNC:52042]
MTRNR2L12	0.01	-1.8	down	1.2	up	-1.1	down	MT-RNR2-like 12 [Source:HGNC Symbol;Acc:HGNC:37169]
MTRNR2L8	0.02	-2.7	down	-1.1	down	-1.3	down	MT-RNR2-like 8 [Source:HGNC Symbol;Acc:HGNC:37165]
RPL14P3	0.03	-1.9	down	-1.3	down	1.0	up	ribosomal protein L14 pseudogene 3 [Source:HGNC Symbol;Acc:HGNC:36638]
RPS18	0.01	-2.3	down	-2.1	down	1.1	up	ribosomal protein S18 [Source:HGNC Symbol;Acc:HGNC:10401]
RPS3AP47	0.04	-2.1	down	-1.8	down	1.0	up	ribosomal protein S3a pseudogene 47 [Source:HGNC Symbol;Acc:HGNC:36744]
SNORD58B	0.02	-1.7	down	-1.6	down	1.1	up	small nucleolar RNA, C/D box 58B [Source:HGNC Symbol;Acc:HGNC:10209]
TRGV4	0.04	-1.6	down	-2.7	down	-2.2	down	T cell receptor gamma variable 4 [Source:HGNC Symbol;Acc:HGNC:12289]

FC = Fold change, ND= Nicotine dependence.

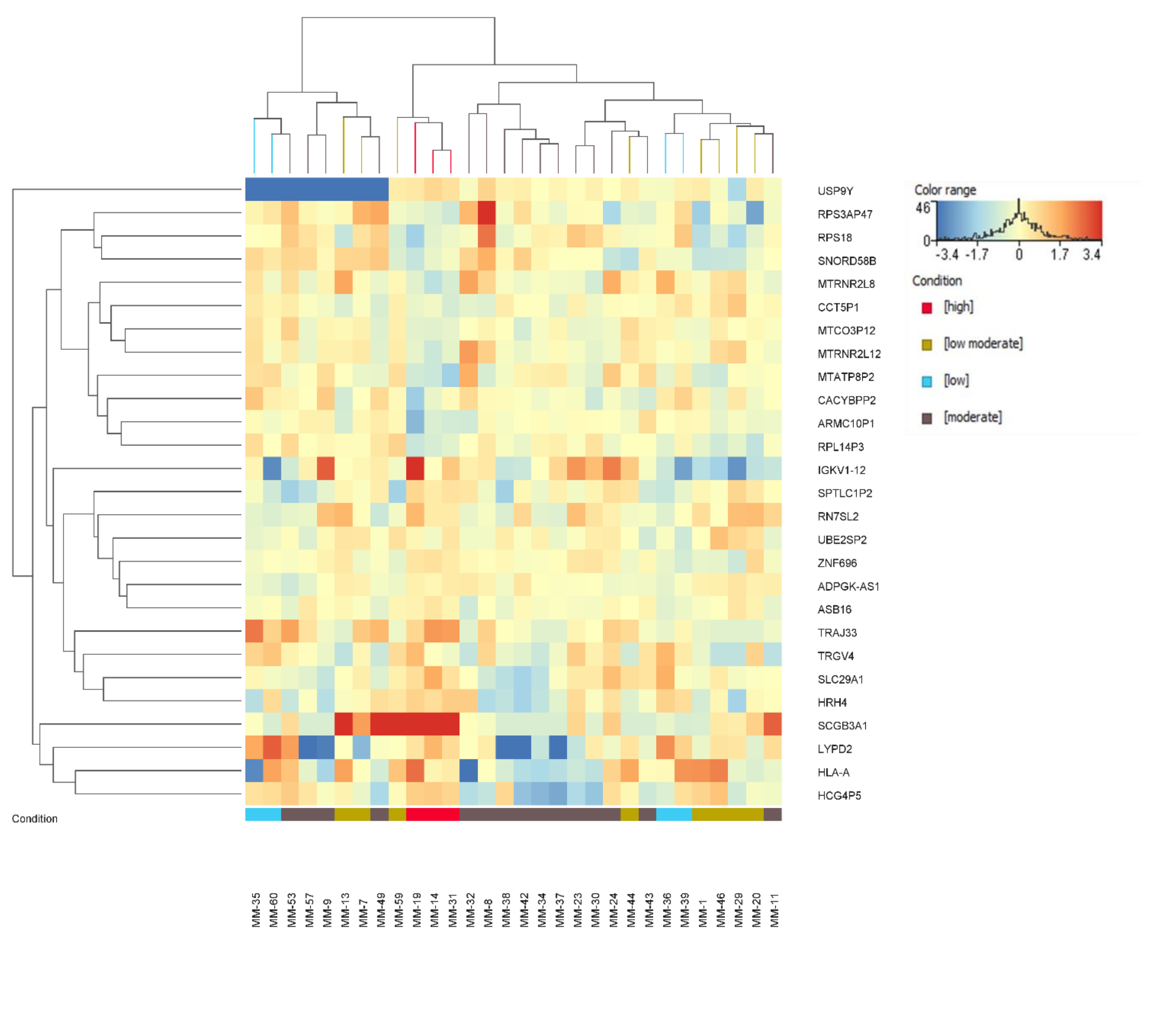

Figure S2. The heatmap of the DEGs among smokers based on nicotine dependence (n=31).

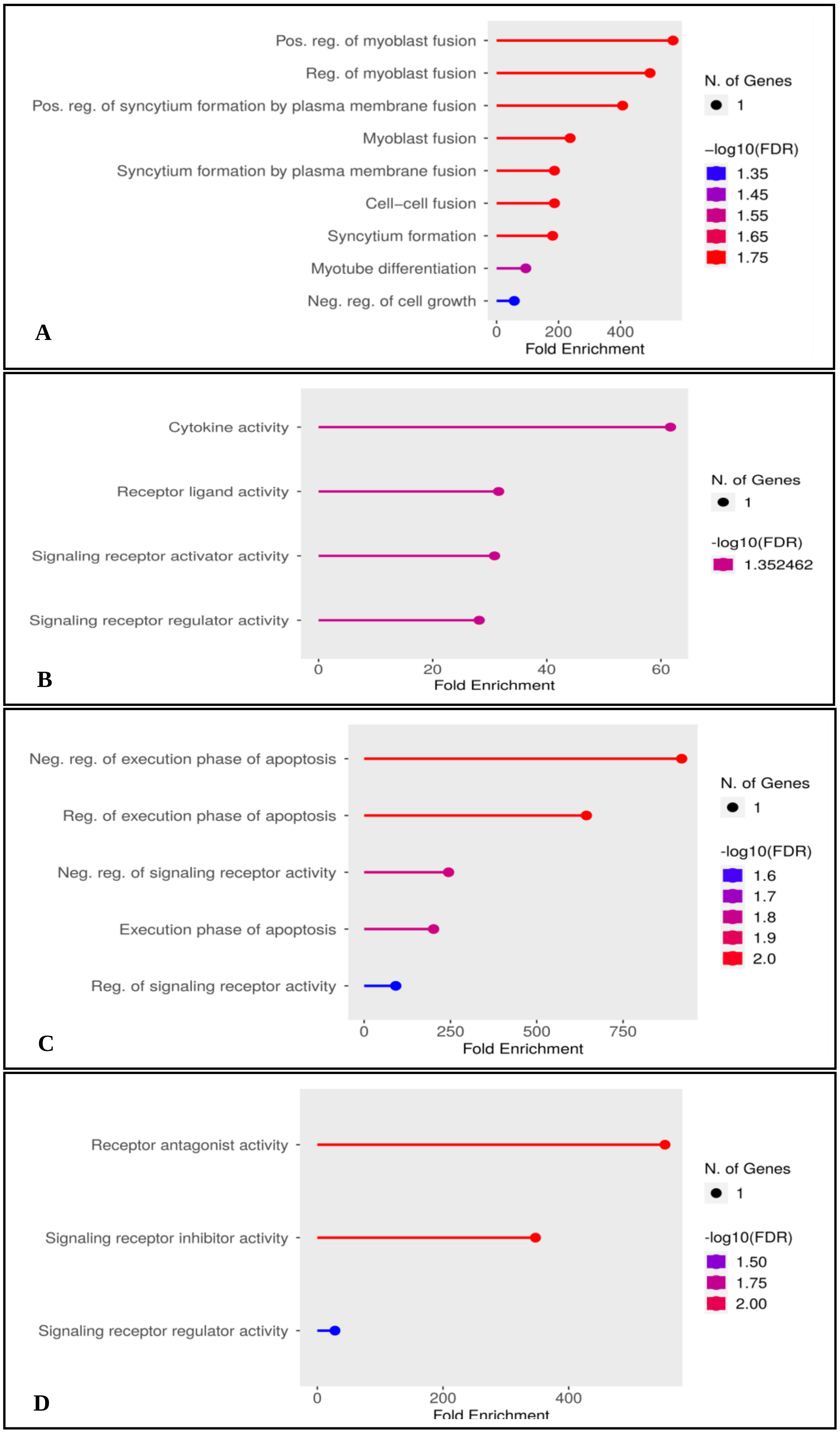

The identified DEGs were based on comparing all nicotine dependence (ND) categories with the low ND.

(A) Significant biological processes of the upregulated genes;
(B) Significant molecular functions of the upregulated genes;
(C) Significant biological processes of the downregulated genes;
(D) Significant molecular functions of the downregulated.

Figure S3. GO analysis of the DEGs among smokers categorized based on nicotine dependence.
